# Nasty and Noble Notes: Interdependence Structures Drive Self-Serving Gossip

**DOI:** 10.1177/01461672231171054

**Published:** 2023-05-25

**Authors:** Terence D. Dores Cruz, Romy van der Lee, Myriam N. Bechtoldt, Bianca Beersma

**Affiliations:** 1 Vrije Universiteit Amsterdam, The Netherlands; 2 EBS Universität für Wirtschaft und Recht, Oestrich-Winkel, Germany

**Keywords:** gossip, trustworthiness, interdependence, prosocial, proself

## Abstract

Much information people receive about others reaches them via gossip. But is this gossip trustworthy? We examined this in a scenario study (*N*_senders_ = 350, *N*_observations_ = 700) and an interactive laboratory experiment (*N*_senders_ = 126; *N*_observations_ = 3024). In both studies, participants played a sequential prisoner’s dilemma where a gossip *sender* observed a *target*’s (first decider’s) decision and could gossip about this to a *receiver* (second decider). We manipulated the interdependence structure such that gossipers’ outcomes were equal to targets’ outcomes, equal to receivers’ outcomes, or independent. Compared to no interdependence, gossip was more often false when gossipers were interdependent with targets but not when interdependent with receivers. As such, false positive gossip (self-serving when interdependent with targets) increased but false negative gossip (self-serving when interdependent with receivers) did not. In conclusion, the interdependence structure affected gossip’s trustworthiness: When gossipers’ outcomes were interdependent with targets, gossip was less trustworthy.

A large share of the information people receive about others reaches them via gossip (Dores Cruz, Thielmann, et al., 2021; Robbins & Karan, 2019), defined as a sender communicating to a receiver about an absent target (Dores Cruz, Nieper, et al., 2021). Gossip provides people with access to more information about others than would be accessible based on direct observation (e.g., Dunbar, 2004; Sommerfeld et al., 2007) and enables people to behave differently toward targets of positive gossip (e.g., helping, cooperating, or befriending) compared to negative gossip (e.g., avoiding, excluding, or punishing; Archer & Coyne, 2005; Dores Cruz et al., 2019; Hess & Hagen, 2019; McAndrew, 2019; Wu et al., 2016b). Although gossip thus provides people with a rich source of information that allows them to navigate their social environment, there is one caveat: Gossip may contain biased or false information (e.g., Fonseca & Peters, 2021; Giardini et al., 2022; Hess & Hagen, 2006a; Peters & Fonseca, 2020). This creates a dilemma for receivers: Should they trust gossip and rely on it when deciding how to behave toward targets? Or should they distrust gossip, ignore it, or behave in ways that are opposed to what they would do if the gossip were true?

While lay perceptions of gossip often depict it as an untrustworthy information source (Dores Cruz, Nieper, et al., 2021), the current scientific literature on gossip cannot answer whether gossip can be trusted (see also Giardini et al., 2022). This is because research on gossip has been conducted within two unconnected streams of literature. The first views gossip as “noble”, prosocial behavior that is driven by the *desire to benefit others* and therefore (often implicitly) views gossip as trustworthy, useful, and reliable (Beersma et al., 2019; Feinberg et al., 2012, 2014; Giardini et al., 2022; Lee & Barnes, 2021; Nowak & Sigmund, 2005; Sommerfeld et al., 2007). In contrast, the second stream of literature views gossip as “nasty” proself behavior that is driven by the *desire to benefit oneself* (potentially via false gossip) and therefore considers gossip to be untrustworthy and unreliable (Beersma et al., 2019; Buss & Dedden, 1990; Giardini et al., 2022; Hess & Hagen, 2006a; Lee & Barnes, 2021; McAndrew et al., 2007; Peters & Fonseca, 2020).

We integrate these two disparate perspectives by considering the context in which gossip occurs. As an inherently social behavior, a key context for understanding gossip is formed by the relationships between the people in the gossip triad: the sender, receiver, and target (Dores Cruz, Nieper, et al., 2021; Giardini et al., 2022; Wittek & Wielers, 1998). These relationships result in an *interdependence structure* that reflects the degree to which parties can influence each other’s outcomes (Balliet et al., 2016; Wu et al., 2021).^1^ Gossip senders’ interdependence with gossip targets and gossip receivers may be crucial for understanding their behavior because it captures the costs and benefits that can drive their behavior in a way that avoids costs and reaps benefits. Recent field research and models indeed suggest that interdependence structures are crucial for shaping how people use gossip (Dores Cruz, Thielmann, et al., 2021; Wu et al., 2021).

Building on this, a striking difference between the two streams of literature discussed above is that gossip has been examined within different interdependence structures. While studies demonstrating prosocial motives have examined gossip within contexts characterized by no interdependence (i.e., parties’ outcomes are uncorrelated), studies demonstrating proself motives for gossip have examined gossip in contexts in which senders were interdependent with receivers or targets (i.e., parties’ outcomes were correlated). As such, it is possible that different interdependence structures can bring to the fore either prosocial or proself motives to gossip. Therefore, we propose that these different interdependence structures may also increase or decrease the likelihood that gossip contains truthful information. We outline how interdependence between the sender and the receiver or target of gossip should increase proself gossip, while no interdependence between the sender and receiver or target should inhibit proself gossip. Systematic experimental investigation of the interdependence structure underlying gossip allows us to draw conclusions about how the trustworthiness of gossip varies across different interdependence structures.

## Gossip for “Noble” Prosocial Motives and the Interdependence Structures Driving It

Many studies have pointed to gossip as a “noble” prosocial behavior (Beersma & Van Kleef, 2012; Dores Cruz et al., 2019; Engelmann et al., 2016; D. Fehr & Sutter, 2019; Fonseca & Peters, 2018; Milinski, 2019; Peters & Fonseca, 2020; Sommerfeld et al., 2007; Wu et al., 2016a, 2016b). People use opportunities to gossip or indicate a strong desire to do so when this can benefit others, which often involves sharing true information. For example, Feinberg et al. (2012) found that participants who observed a norm violation gossiped to protect receivers from exploitation by the norm violator in an upcoming interaction, even when this could not lead to punishment of the norm violator and when they had to incur costs to gossip.

Closely examining the studies underlying the conclusion that gossip is driven by prosocial motives shows that these studies have commonly investigated gossip in contexts where gossip can be shared with and about parties whose outcomes cannot influence the gossiper’s outcomes. This brings forward the “noble” side of gossip because it creates a situation in which protecting others from becoming a victim or promoting cooperation between others is salient (Dores Cruz et al., 2019). As such, studying gossip within a context involving no interdependence between the sender and other parties is likely to lead to the conclusion that gossip is noble and prosocial behavior.

## Gossip for “Nasty” Proself Motives and the Interdependence Structures Driving It

In contrast, other studies have pointed to gossip as a “nasty” proself behavior (Archer & Coyne, 2005; Davis et al., 2019; Hess & Hagen, 2006b, 2019, 2021; Massar et al., 2012; McAndrew, 2017; McAndrew et al., 2007; Wittek & Wielers, 1998). People have frequently been found to gossip or indicate a high willingness to do so when this can benefit themselves, which often involves sharing false or manipulated information. For example, Hess and Hagen (2021) had participants read vignettes that involved competition for resources (e.g., a single promotion with a pay raise). Participants were more likely to gossip negatively about their rivals if it helped them compete successfully, and their willingness to gossip increased when the resource was scarcer and more valuable.

Closely examining the studies underlying the conclusion that gossip is driven by proself motives shows that these studies have commonly investigated gossip in contexts where it can be shared with and about parties whose outcomes can influence the gossiper’s outcomes. This interdependence context brings forward the “nasty” side of gossip because it creates a situation in which benefiting oneself through gossip is in the foreground (Dores Cruz et al., 2019). As such, studying gossip within a context involving interdependence between senders and other parties is likely to lead to the conclusion that gossip is nasty and proself behavior.

## Integrating Prosocial and Proself Motives Through the Interdependence Context

We argue that people are sometimes motivated to gossip for prosocial reasons, and at other times for proself reasons (Dores Cruz et al., 2019; Hess & Hagen, 2021; McAndrew, 2019). Specifically, when the gossiper’s outcomes are independent of the receiver or target, it is unlikely that proself motives drive gossip. In such instances, the motivation to gossip could be to protect others from exploitation (e.g., Beersma & Van Kleef, 2012; Feinberg et al., 2012; Milinski, 2019). Furthermore, when the gossiper and the receiver or target are interdependent, proself motives more likely drive gossip because, in this case, gossip can lead to benefits for gossipers (e.g., Hess & Hagen, 2019, 2021; McAndrew, 2019; McAndrew et al., 2007).

If different interdependence structures trigger prosocial versus proself motives, it is likely that interdependence structures also affect the extent to which gossip is trustworthy. Within interdependence structures triggering prosocial motives, gossip senders will likely transmit truthful information.^2^ Within interdependence structures triggering proself motives, gossip will be more likely false because manipulating others can serve proself motives. Accordingly, gossip is likely to be more trustworthy when gossipers are independent and less trustworthy if they are interdependent. Yet, in the two streams of literature discussed earlier, gossip was studied within a single interdependence structure and the interdependence structure usually remained implicit (Giardini & Wittek, 2019). Therefore, it remains unclear to what extent gossip can be trusted across different interdependence structures.

A recent experimental study on accurate and inaccurate gossip provided initial support for the influence of interdependence structures on the trustworthiness of gossip (Peters & Fonseca, 2020). In the study, gossip senders were either part of a group that competed with another group or a group that did not compete. In this way, it examined whether negative interdependence (i.e., negatively correlated outcomes) between gossip senders and receivers (from a competing group) would elicit inaccurate gossip. Selfish lies (distorting information to benefit one’s own group) formed a minority of gossip when senders and receivers were independent but became more frequent under negative interdependence.

Although these results provide initial evidence that interdependence structures can drive proself false gossip, they do not take into account that such gossip can take different forms depending on whether targets were cooperative or uncooperative and whether gossipers benefit from a receiver or a target maximizing their outcomes. On the one hand, proself gossip can manifest itself in misrepresenting an uncooperative target as cooperative so that the receiver could decide to reciprocate the alleged cooperation. In this case, gossip would contain false positive information about a target. This would be a rational strategy if a gossiper benefited from the target maximizing their outcomes by getting the receiver to cooperate and thereby be exploited by an uncooperative target. On the contrary, there are also contexts in which a gossiper could benefit from misrepresenting a cooperative target as uncooperative so that the receiver could decide to reciprocate the alleged defection. In this case, gossip would involve false negative information about a target. This would be a rational strategy if a gossiper benefited from the receiver maximizing their outcomes by getting the receiver not to cooperate with the target (i.e., defecting and exploiting the target).

Moreover, gossiping is not the only way to behave selfishly. People may be driven to refrain from gossiping by proself motives, just as they may be driven to gossip by proself motives: Not informing someone about a third party’s behavior when one could benefit from it can also be considered as “nasty.” This raises the question of whether people prefer to gossip or refrain from gossiping. This question has received little attention and previous studies cannot answer it because, in experiments, gossiping was either often mandatory (e.g., Giardini & Wittek, 2019; Peters & Fonseca, 2020; Sommerfeld et al., 2007), or simulations and field studies focused on shared gossip but not on refraining from gossip (e.g., Dores Cruz, Thielmann, et al., 2021; Wu et al., 2021).

Interdependence structures may also affect refraining from gossip (Giardini & Wittek, 2019). For example, potential gossip senders can benefit themselves by sharing negative (positive) gossip about rivals (allies) but refrain from sharing such gossip about allies (rivals). Similarly, refraining from negative gossip can benefit a target and harm a receiver whereas refraining from positive gossip can harm receivers and targets.

## The Current Study

**Figure 1.**
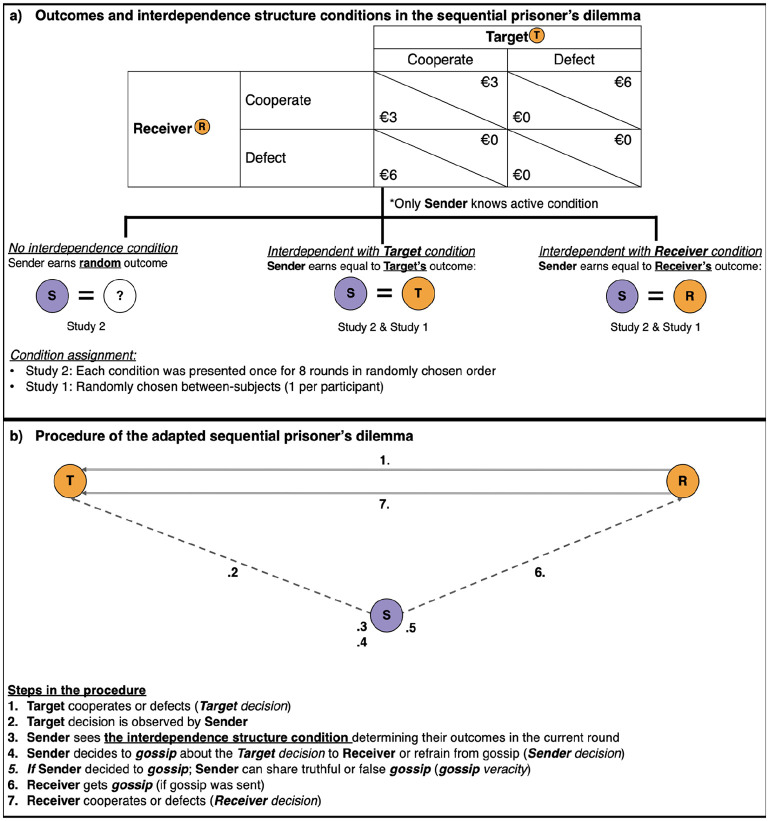
Overview of All the Steps (Numbers 1–7) in the Adapted Sequential Prisoner’s Dilemma Including (a) the Outcomes Per Interdependence Structure Condition in the Sequential Prisoner’s Dilemma, and (b) the Procedure of the Game. *Note*. Outcomes in Study 1 were $50 instead of 3€, 100$ instead of 6€, and 10$ for each if both players chose to defect instead of 0€ (making the game a standard prisoner’s dilemma instead of a weak prisoner’s dilemma).

We conducted an online scenario study (Study 1) and a preregistered interactive lab experiment (Study 2). For these studies, we adapted a sequential prisoner’s dilemma game (Clark & Sefton, 2001; Rapoport, 1988) to create a context for gossip. At the beginning of the game, a first decider chose whether to cooperate with a second decider or not. After the first decider’s choice a second decider chose whether to cooperate with the first decider or not. We added a person who observed the choice of the first decider. This observer chose whether or not to gossip about the first decider’s decision; thus, the observer was the gossip *sender*, the first decider the gossip *target* and the second decider the gossip *receiver*. In case the sender chose to gossip to the receiver, the sender could either gossip truthfully or falsely. The second decider received this information before deciding to cooperate or defect (see Figure 1).

We manipulated the interdependence structure by varying how the outcomes of the sender were determined. We examined two interdependence structure conditions: In the *interdependent with the target* condition, the sender obtained the same outcomes as the target. In the *interdependent with the receiver* condition, the sender obtained the same outcomes as the receiver. In both conditions, senders could gossip to manipulate receivers into acting in their favor. In Study 2, we added a *no interdependence condition*, where the sender’s outcomes were independent of others’ outcomes.

We built our hypotheses on theory and research findings on reciprocal cooperation. Reciprocity entails that cooperation is responded to with cooperation or other benefits and, similarly, that a person’s uncooperative acts (e.g., defecting in the prisoner’s dilemma) are responded to with non-cooperation or other costs (e.g., Nowak & Sigmund, 2005; Wu et al., 2016b). As such, even though it would be rational from a gossip receiver’s individual perspective to respond with defection to cooperation of the first decider to maximize personal outcomes in our studies, we assumed that most receivers follow the norm of reciprocity and display reciprocal cooperation. Moreover, we assumed that gossipers would expect receivers to reciprocate the alleged or true (un)cooperative behavior of a target (leading either to mutual cooperation/defection if gossip was true or to one party cooperating and the other defecting if gossip was false).

A plethora of studies supports theorizing on reciprocity. People have a strong desire to follow a norm of reciprocity that entails cooperating for mutual gains with others that are known to be cooperative and thus refrain from exploiting cooperation even if that is individually more beneficial (e.g., E. Fehr & Fischbacher, 2003; Romano et al., 2022; Yamagishi et al., 2014). To illustrate, in a sequential prisoner’s dilemma where second deciders know whether first deciders cooperated or defected before making their decision, the second deciders are likely to reciprocate the first decider’s cooperation and defection (Clark & Sefton, 2001; Eichenseer & Moser, 2020; Khadjavi & Lange, 2013). With regard to gossip, receivers have been shown to use gossip to guide their reciprocal cooperation: Receivers are likely to respond with (un)cooperative behavior to (un)cooperative behavior of targets (Dores Cruz, Thielmann, et al., 2021; Fonseca & Peters, 2021; Milinski, 2019; Sommerfeld et al., 2007; Wu et al., 2016b).

We reasoned that senders would take these tendencies toward reciprocation into account when gossiping and that, therefore, the two conditions in which senders were interdependent with the receiver or target would motivate two types of self-serving false gossip. First, if the target defected and the sender’s outcomes are dependent on the target’s outcomes, the sender should be more likely to falsely describe the target as cooperative than when senders and targets have no interdependence (Hypothesis 1 [H1]). Second, if the target cooperated and the sender’s outcomes are dependent on the receiver’s outcomes, the sender should be more likely to falsely describe the target as uncooperative than when senders and receivers have no interdependence (Hypothesis 2 [H2]). On this basis, we expected both interdependence conditions to cause gossip to be less trustworthy (contain more false information). We also tested exploratively when participants (strategically) decide to refrain from gossiping as well as whether receiving no gossip, positive gossip, or negative gossip impacts receivers’ decisions to cooperate or defect.

## Study 1

Based on the hypotheses outlined above, we expected participants to be more likely to gossip falsely if they could benefit from doing so.^3^ To test whether interdependence structures elicited the expected false gossip, we compared the prevalence of untrustworthy gossip that could benefit senders to untrustworthy gossip that could not. In the *interdependent with the target* condition, false positive gossip (describing a target that defected as having cooperated) could benefit senders (if the receiver reciprocates the alleged cooperation), and this is expected to be more prevalent than false negative gossip (describing a target that cooperated as having defected). In the *interdependent with the receiver condition*, false negative gossip could benefit senders (if the receiver reciprocates the alleged defection) and is expected to be more prevalent than false-positive gossip.

### Method

We preregistered this study (https://osf.io/3b478), and data, code, and materials are openly accessible (https://osf.io/dkjnh).

#### Participants

A total of 380 participants were recruited using Amazon’s Mechanical Turk. Thirty participants were excluded for failing attention checks and/or providing nonsensical answers to open questions. The final sample consisted of 350 participants (56.6% female) aged 19 to 71 years (*M* = 36.84, *SD* = 10.54). Participants were paid $4.

##### Power Analysis

We conducted a priori power analysis to detect a small to medium effect (*w* = 0.15) with 80% power using G*Power and *α* = .05 (Faul et al., 2007), which indicated that a total sample size of 349 participants was required.

#### Procedure and Materials

All participants signed an informed consent form and were instructed that they would engage in a hypothetical interaction task involving two others (adapted sequential prisoner’s dilemma game; Clark & Sefton, 2001; Rapoport, 1988). Although the decisions were hypothetical and did not affect any payoffs, participants were asked to respond as if they were making actual decisions. Participants were randomly assigned to one of the two interdependence structure conditions when they acted as the sender. They were informed about the structure of the adapted sequential prisoner’s dilemma game (see Figure 1). If both deciders in the prisoner’s dilemma cooperated, each would hypothetically receive $50. If one cooperated, while the other defected, the cooperator received nothing, and the defector received $100. If both defected, each received $10. In the game, gossip targets would decide first to cooperate or defect (*target decision*). After this, the potential gossip senders learned about the targets’ decisions and could choose to gossip or not. Finally, the receivers would make their decision without knowing what the target had actually done.

After answering comprehension questions, participants started the game. We asked participants to imagine how they would behave in each of the three roles; participants first acted as the target, then as the receiver, and finally as the sender. After making their decision in each role, participants provided demographic information, were debriefed, and paid. We did not use the word “gossip” to avoid negative connotations (e.g., Dores Cruz, Thielmann, et al., 2021) but asked if the senders wanted to “share information.” We report results regarding participants’ decisions as senders and receivers.

To examine gossip, we employed a strategy method, which implies that participants decided to gossip for each possible target decision (cooperate or defect presented below one another simultaneously; “Please indicate if you want to share information about the target to the receiver” [yes, no]). If they chose to gossip, participants wrote a free-text message about the target’s decision followed by indicating on a forced-choice item whether they would want to dichotomously gossip that the target cooperated or defected or if they did not want to send only such dichotomous gossip (there were 59 observations of the latter, these were excluded from analyses on gossip veracity).^4^ To assess gossip veracity, we compared the answer gossip senders gave on the forced-choice item with the hypothetical target’s decisions. When gossip matched the target’s decision, it was rated as true; otherwise, it was rated as false. After making their decisions as senders, participants completed self-report items about how their gossip could impact themselves and others (see Supplemental Material). The potential gossipers were presented with a table detailing the prisoner’s dilemma and their possible outcomes, which provided information on the interdependence structure condition, and were informed that only the senders would know how the outcomes of senders were determined.

Gossip was hypothetical and not sent to a receiver. Instead, participants themselves acted as gossip receivers using a strategy method. When participants acted as receivers, their hypothetical outcomes were determined by another alleged participant who would act first and they made their decision to cooperate or defect in return (*receiver decision; see Supplemental Material*). As in the strategy method for gossip, participants made their decision about cooperation or defection separately for all possible types of gossip: gossip about the target cooperating, gossip about the target defecting, and no gossip. They were presented with a table detailing the prisoner’s dilemma game including their possible outcomes.

#### Statistical Analyses

First, to analyze gossip veracity (0 = *true*, 1 = *false*) and the sender’s decision to gossip (0 = *no*, 1 = *yes*), we used generalized (binomial) mixed models with fixed effects for the interdependence structure condition (0 = *interdependence with the receiver*, 1 = *interdependence with the target*), and target decision (0 = *defect*, 1 = *cooperate*) and the interaction between interdependence structure condition and target decision as the independent variables. Within each condition, we compared false gossip that could be self-serving with false gossip that could not be self-serving. The model included random intercepts for participants (we report results without control variables; results did not differ when controlling for age and gender, see Supplemental Material).

### Results

Participants decided to gossip more often than not (67.1%), which resulted in 470 observations of gossip (each participant could gossip about cooperation and/or defection). Table 1 presents an overview of gossip across interdependence structure conditions and target decisions. Table 2 presents an overview of when senders gossiped truthfully or falsely which shows that, overall, the majority of gossip was trustworthy (84.7%).

**Table 1.** Frequency of Gossip Depending on Conditions and Target Decisions (Study 1).

Target decision	Interdependence structure condition					
	Interdependent with the receiver		Interdependent with the target		Overall	
	Gossip	No gossip	Gossip	No gossip	Gossip	No gossip
Cooperate	141 (80.6%)	34 (19.4%)	114 (65.1%)	61 (34.9%)	255 (72.9%)	95 (27.1%)
Defect	136 (77.7%)	39 (22.3%)	79 (45.1%)	96 (54.9%)	215 (61.4%)	135 (38.6%)
Overall	277 (79.1%)	73 (20.9%)	193 (55.1%)	157 (44.9%)	470 (67.1%)	230 (32.9%)

**Table 2.** Frequency of True Versus False Gossip Across Conditions and Target Decisions (Study 1).

Target decision	Interdependence structure condition					
	Interdependent with the receiver		Interdependent with the target		Overall	
	True gossip	False gossip	True gossip	False gossip	True gossip	False gossip
Cooperate	122 (96.1%)	5 (3.9%)	75 (78.9%)	20 (21.1%)	197 (88.7%)	25 (11.3%)
Defect	106 (87.6%)	15 (12.4%)	45 (66.2%)	23 (33.8%)	151 (79.9%)	38 (20.1%)
Overall	228 (91.9%)	20 (8.1%)	120 (73.6%)	43 (26.4%)	348 (84.7%)	63 (15.3%)

**Figure 2.**
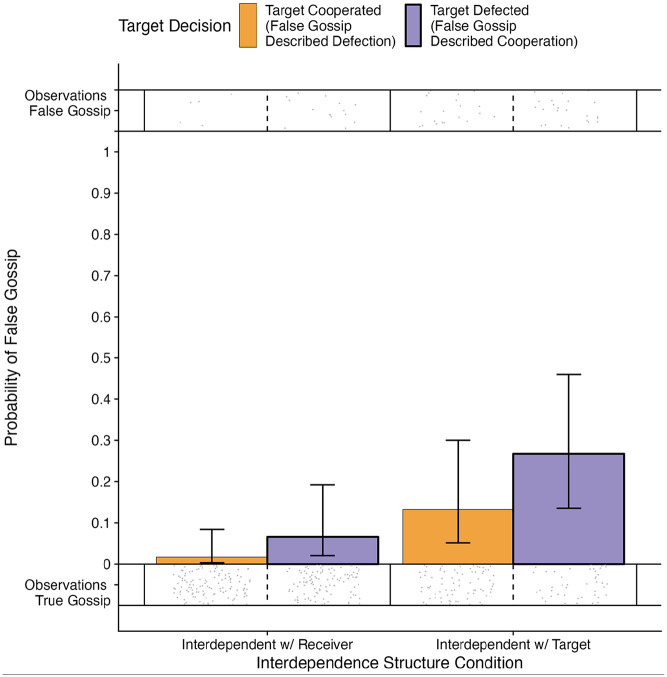
The Predicted Probability to Engage in False Gossip Per Interdependence Structure Condition and Target Decision (Study 1). *Note*. The error bars indicate a 95% confidence interval. The dots indicate individual observations of true gossip and false gossip.

In the interdependent with the target condition, the difference between self-serving false positive gossip (i.e., describing a defecting target as having cooperated) and false negative gossip that was unable to be self-serving (i.e., describing a cooperating target as having defected) trended in the expected direction of self-serving false gossip occurring more often. Yet the difference was not statistically significant using an *α* of .05: odds ratio [*OR*] = 2.39, standard error [*SE*] = 1.11, 95% confidence interval [CI]: [0.97, 5.92], *z* = 1.88, *p* = .060. There were few observations of false gossip which could hamper detecting (small) differences in the proportion of false gossip, and it is noteworthy that the effect size indicates that false gossip was more likely when participants were interdependent with the target and the target had defected (see Figure 2 and Table 2).

In the interdependent with the receiver condition, self-serving false negative gossip (i.e., describing a cooperating target as having defected) was not more likely than false positive gossip that was unable to be self-serving (i.e., describing a defecting target as having cooperated). In fact, self-serving false gossip was significantly less likely than false gossip that was not self-serving (*OR* = 0.25, *SE* = 0.15, 95% CI [0.08, 0.78], *z* = −2.38, *p* = .017).

### Discussion

Participants in the interdependent with the target condition were not significantly more likely to share untrustworthy gossip that could benefit themselves compared to untrustworthy gossip that could not. However, the effect size and the descriptive pattern of untrustworthy gossip did indicate that self-serving false positive gossip occurred more frequently. Participants in the interdependent with the receiver condition were also not significantly more likely to share untrustworthy gossip that could benefit themselves compared to untrustworthy gossip that could not. Thus, participants rarely used false negative gossip even if it could benefit them.

Self-report items indicated that for some participants, gossip was driven by selfish motives (see Supplemental Material), and for participants who sent false gossip, false positive gossip was more frequent than false negative gossip. However, this difference could be intertwined with the incentive structure of the prisoner’s dilemma game. That is, false positive and false negative gossip was financially differentially attractive to gossipers if they expected their gossip to be responded to with reciprocal behavior by the receiver: False positive gossip could lead to a benefit of €100 whereas false negative gossip could lead to a benefit of €50; we return to this issue in the General Discussion. Therefore, we did not statistically test the difference between the two conditions. Because this inequality in incentives cannot be remedied without changing the structure of the prisoner’s dilemma game, we instead addressed this limitation in the following experiment by comparing these different types of false gossip in the interdependence conditions with a condition involving no interdependence. This provides insight into how incentive structures affect these two types of false gossip and sheds light on whether interdependence elicits more untrustworthy selfish gossip than no interdependence.

Another key limitation is that the scenario used in Study 1 was a single shot, unincentivized, and non-interactive. This prevents us from drawing strong conclusions about whether people would actually engage in gossip and perhaps send false gossip if that actually impacted outcomes. Moreover, Study 1 yielded few observations of gossip in contexts relevant to self-serving false gossip, reducing statistical power. We addressed those limitations in Study 2 as well.

## Study 2

Study 2 aimed to test whether interdependence structures would impact the trustworthiness of gossip in an interactive direct response design using multiple rounds to create more opportunities to use gossip strategies, increase statistical power, and incorporate actual monetary earnings. As described above, we added a no interdependence condition in which senders’ outcomes were independent of others’ outcomes. This reflects the literature on prosocial gossip. No interdependence should elicit more trustworthy or prosocial gossip because gossipers can only affect others’ outcomes and not their own. Therefore, we expected interdependence with targets and receivers to reduce gossip’s trustworthiness compared to no interdependence.

### Method

We preregistered this study (https://osf.io/9hfbm) and data, code, and materials are openly accessible (https://osf.io/af4rc).

#### Participants

A total of 378 students,^5^ 126 participants per role (first decider/target, second decider/receiver, observer/sender) were recruited on the campus of the Vrije Universiteit Amsterdam (a large public university in the Netherlands). Gossip senders were 44 men (34.9%) and 82 women (65.1%), and their ages ranged from 18 to 32 years (*M* = 20.71, *SD* = 2.48). Gossip receivers were 37 men (29.37%) and 89 women (70.63%), their ages ranged from 18 to 41 years (*M* = 20.35, *SD* = 2.68). Gossip targets were 39 men (30.95%) and 86 women (68.25%), and one participant indicated other (0.79%); their ages ranged from 18 to 27 years (*M* = 20.71, *SD* = 2.14). All participants played 24 rounds of the experiment, resulting in 3024 observations per role. Participants were paid €12 and could earn a bonus of up to €6 (a randomly selected round was paid out).

##### Power Analysis

We conducted a power analysis to test a three-level (rounds, interdependence structure condition, participants) model with a binary outcome using MLPOWSIM (Browne et al., 2009). The simulation indicated that 126 participants playing the role of gossip sender provide 80% power to detect small to medium effects when using three conditions and eight rounds per condition (requiring 126 targets and 126 receivers to interact with; total of 378 participants).

#### Procedure and Materials

The procedure was largely identical to Study 1 with four exceptions. First, participants truly interacted with each other in 24 rounds of the game which would determine their bonus. In each round, participants were paired in different groups of 3, with one participant per role. Participants were randomly assigned to the role of the gossip target, sender, or receiver. Participants remained in the same role throughout all 24 rounds. Participants took part in sessions of 9 people where an experimenter gave general and role-specific instructions. Participants were not allowed to talk to each other and could only ask the experimenter questions during the instruction phase.

Second, we added a no interdependence condition in which gossipers’ outcomes were randomly determined (€0–€6), removing the incentive for the sender to communicate false gossip because the receiver’s decision did not impact the sender’s outcomes. In random order, eight rounds were played in one of three *interdependence structure conditions.* Receivers and targets knew of the possible interdependence structure conditions but only the sender knew the actual condition in a given round. Participants did not receive feedback about the outcomes of a round, they only learned of the outcomes of the randomly selected round that would be paid as a bonus at the end of the study.

Third, we used the structure of a weak, instead of a standard, prisoner’s dilemma game. A weak prisoner’s dilemma has similar properties to the standard prisoner’s dilemma used in Study 1, except for the outcome when one decider cooperates while the other decider defects (known as the “sucker outcome” for the exploited cooperating decider) is the same as the outcome resulting from when both deciders defect (known as the “punishment outcome,” Rapoport, 1988).^6^ We used this weak prisoner’s dilemma with the aim of creating a more equal distribution of (real) targets’ cooperation and defection which the senders could gossip (or remain silent) about, as previous research indicates cooperation could be more prevalent in the weak prisoner’s dilemma (Rapoport, 1988). Specifically, if both targets and receivers cooperated, each received €3. If one cooperated while the other defected, the cooperator received nothing, and the defector received €6. If both defected, each received nothing.

Finally, we modified the question about willingness to gossip. Potential senders now answered two consecutive forced-choice items (“Please indicate if you want to share information about the target to the receiver” [yes, no]; if yes, please indicate what you want to pass on to the receiver [target chose to cooperate, target chose to defect]). They also had to write a free-text message that would accompany the gossip. Again, when the forced-choice gossip matched the target’s decision, it was rated as true; otherwise, it was rated as false. The receivers were presented with a table detailing the prisoner’s dilemma game including their possible outcomes. If senders decided to gossip to the receiver, the preformulated message of the forced-choice gossip item (i.e., the target chose to cooperate/defect) would be presented along with the free-text message next to the table. For an overview, see Figure 1. In contrast to gossip being hypothetical in Study 1, in each round, a receiver got one gossip message from the sender in their group.

#### Statistical Analyses

To analyze gossip veracity (0 = *true*, 1 = *false*) and sender decision to gossip (0 = *no*, 1 = *yes*), we used generalized (binomial) mixed models with fixed effects for the interdependence structure condition (0 = *no interdependence*, 1= *interdependent with the target*, 2 = *interdependent with the receiver*), and target decision (0 = *defect*, 1 = *cooperate*) and the interaction between these variables as independent variables as the independent variables. We used contrast analyses to test H1 and H2 and compare false gossip within conditions as for Study 1. We further used a generalized (binomial) mixed model with fixed effect for the gossip event (0 = *no gossip received*, 1= *received gossip about cooperation*, 2 = *received gossip about defection*) as the independent variables to analyze the receiver’s reactions to gossip (1 = *cooperate*, 0 = *defect*). We used pairwise comparisons with a Bonferroni correction to compare gossip events. All models included random intercepts for participants (we report results without control variables, see Supplementary Information for analyses controlling for data collection wave, round number, age, and gender; the results were virtually identical).

### Results

#### Overview of Decision to Gossip and Gossip Trustworthiness

Participants decided to gossip more often than not (66.5%), which resulted in 2010 observations of gossip (see Table 3 for an overview of gossip across interdependence structure conditions and target decisions). Table 4 presents an overview of when senders gossiped truthfully or falsely, which shows that, overall, most gossip was trustworthy (76.2%). However, the trustworthiness of gossip differed between conditions.^7^

**Table 3.** Frequency of Senders Gossiping Across Conditions and Target Decisions (Study 2).

Target decision	Interdependence structure condition							
	Interdependent with the target		Interdependent with the receiver		No interdependence		Overall	
	Gossip	No gossip	Gossip	No gossip	Gossip	No gossip	Gossip	No gossip
Cooperate	282 (62.5%)	169 (37.5%)	407 (71.6%)	161 (28.4%)	292 (63.3%)	169 (36.7%)	942 (69.7%)	410 (30.3%)
Defect	346 (62.1%)	211 (37.9%)	775 (76.9%)	233 (23.1%)	315 (57.6%)	232 (42.4%)	1068 (63.9%)	604 (36.1%)
Overall	628 (62.3%)	380 (37.7%)	368 (83.6%)	72 (16.4%)	607 (60.2%)	401 (39.8%)	2010 (66.5%)	1014 (33.5%)

**Table 4.** Frequency of True Versus False Gossip Across Conditions and Target Decisions (Study 2).

Target decision	Interdependence structure condition							
	Interdependent with the target		Interdependent with the receiver		No interdependence		Overall	
	True gossip	False gossip	True gossip	False gossip	True gossip	False gossip	True gossip	False gossip
Cooperate	232 (82.9%)	48 (17.1%)	328 (89.1%)	40 (10.9%)	257 (88.9%)	32 (11.1%)	817 (87.2%)	120 (12.8%)
Defect	139 (40.6%)	203 (59.4%)	341 (84.0%)	65 (16.0%)	223 (72.2%)	86 (27.8%)	703 (66.5%)	354 (33.5%)
Overall	371 (59.6%)	251 (40.4%)	669 (86.4%)	105 (13.6%)	480 (80.3%)	118 (19.7%)	1520 (76.2%)	474 (23.8%)

#### Hypothesis Tests Regarding Self-Serving False Gossip

The effect of the interdependence structure condition depended on the target decision (Wald χ^2^(2) = 34.45, *p* < .001). Below, we present results for self-serving false gossip when the target defected (false positive gossip misrepresenting defection as cooperation; H1) and for self-serving false gossip when the target cooperated (false negative gossip misrepresenting cooperation as defection; H2).

**Figure 3.**
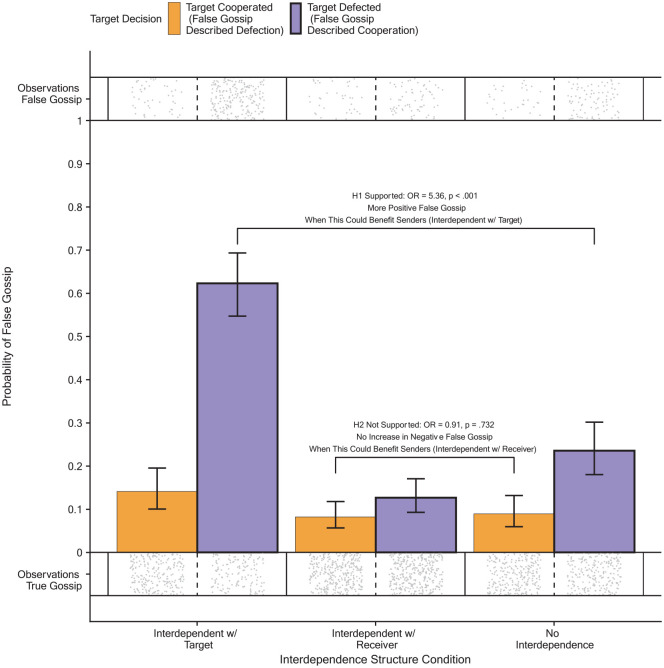
The Predicted Probability to Engage in False Gossip Per Interdependence Structure Condition and Target Decision. The Bracket for H1 Shows Support for H1: When the Target Defected and the Sender Was Interdependent With the Target, False Gossip Describing the Target as Cooperative Was More Likely Compared to When the Sender Had No Interdependence. The Bracket for H2 Shows No Support for H2: When the Target Cooperated and the Sender Was Interdependent With the Receiver, False Gossip Was Not More Likely Compared to When the Sender Had No Interdependence (Study 2). *Note*. The error bars indicate a 95% confidence interval. The dots indicate individual observations of true gossip and false gossip.

In line with H1, contrast analyses showed that senders were more likely to misrepresent an uncooperative target as cooperative in the interdependent with the target condition, where they had an incentive to lie, compared to the no interdependence condition, where they had no incentive to lie (*OR* = 5.36, *SE* = 1.05, 95% CI [3.66, 7.86], *z* = 8.61, *p* < .001). This is depicted in Figure 3. Furthermore, in line with H1, in the interdependent with the target condition, self-serving false positive gossip (i.e., describing a defecting target as having cooperated) was significantly more likely than false negative gossip that was unable to be self-serving (i.e., describing a cooperating target as having defected; *OR* = 10.05, *SE* = 2.24, 95% CI [6.49, 15.55], *z* = 10.35, *p* < .001).

Counter to H2, however, contrast analyses showed that senders were not more likely to misrepresent a cooperative target as uncooperative in the interdependent with the receiver condition, where they had an incentive to lie, compared to the no interdependence condition, where they had no incentive to lie. (*OR* = 0.91, *SE* = 0.24, 95% CI [0.55, 1.53], *z* = −0.34, *p* = .732). This is depicted in Figure 3. Furthermore, counter to H2, in the interdependent with the receiver condition, self-serving false negative gossip (i.e., describing a cooperating target as having defected) was not more likely than false positive gossip that was unable to be self-serving (i.e., describing a defecting target as having cooperated). In fact, self-serving false gossip was significantly less likely than false gossip that was not self-serving (*OR* = 0.62, *SE* = 0.14, 95% CI [0.39, 0.97], *z* = −2.10, *p* = .036).

In the no interdependence condition, false positive gossip was also significantly more likely than false negative gossip (*OR* = 3.15, *SE* = 0.77, 95% CI [1.95, 5.07] *z* = 4.71, *p* < .001).

#### Explorative Analyses Regarding When People Engage in Gossip

**Figure 4.**
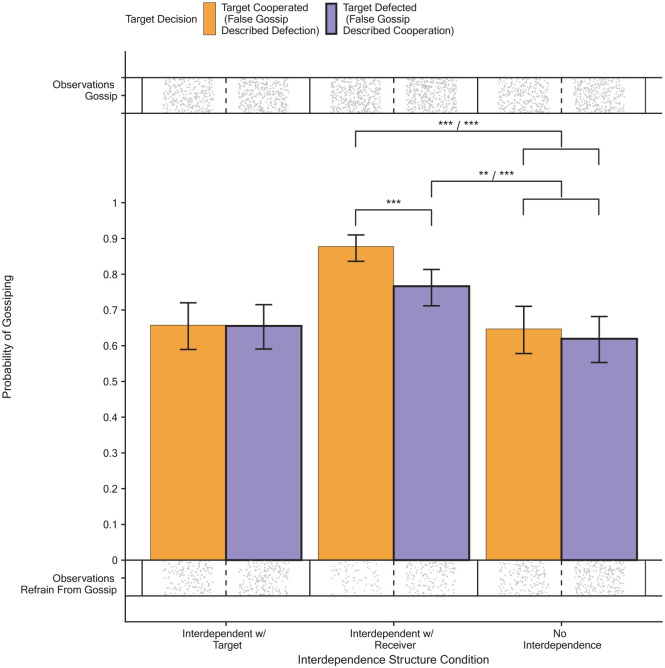
The Predicted Probability to Engage in Any Gossip Per Interdependence Structure Condition and Target Decision. The Brackets Show That Both Gossip About Cooperation and Gossip About Defection Were More Likely When Senders Were Interdependent With the Receiver Compared to When Senders Had No Interdependence (Study 2). *Note*. The error bars indicate a 95% confidence interval. The dots indicate individual observations of true gossip and false gossip. For details of the comparisons, see Supplemental Material.

Whether senders gossiped or not in each condition depended on the target’s decision (see Table 3; *n* = 3024; Wald χ^2^(2) = 12.89, *p* = .002). Figure 4 shows that senders were more likely to gossip in the interdependent with the receiver condition compared to the no interdependence condition. The propensity to gossip did not differ between the interdependent with the target condition and the no interdependence condition. Moreover, in the interdependent with the receiver condition, senders were more likely to gossip when targets cooperated compared to when targets defected (for details, see Supplemental Material).

#### Explorative Analyses Regarding Receivers’ Decisions Following Senders’ Decisions to Gossip or Not

**Figure 5.**
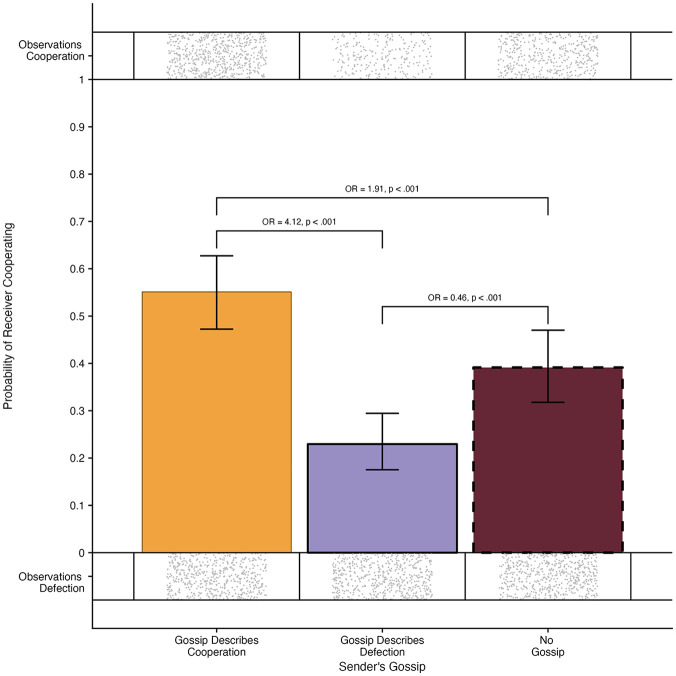
Probability of Cooperation by Receivers When Gossip About Cooperation Was Received, Gossip About Defection Was Received, and When No Gossip Was Received. Receivers Were More Likely to Cooperate When Gossip About Cooperation Was Received and Less Likely To Cooperate When Gossip About Defection Was Received (Study 2). *Note*. The error bars indicate a 95% confidence interval. The dots indicate individual observations of cooperation and defection.

Receivers’ decisions differed based on senders’ gossip behavior, Wald χ^2^(2) = 144.24, *p* < .001 (see Figure 5). Pairwise comparisons showed that receivers who received positive gossip (indicating that the target cooperated) were slightly more likely to cooperate compared to when they did not receive gossip (*OR* = 1.91, *SE* = 0.20, 95% CI [1.48, 2.47], *z* = 6.06, *p* < .001) and moderately more compared to when they received negative gossip (*OR* = 4.12, *SE* = 0.49, 95% CI [3.11, 5.47], *z* = 11.96, *p* < .001). Moreover, receivers that received negative gossip (indicating that the target defected) were slightly less likely to cooperate compared to when they did not receive gossip (*OR* = 0.46, *SE* = 0.06, 95% CI [0.35, 0.62], *z* = −6.34, *p* < .001).

### Discussion

Results partially supported our hypotheses. In line with H1, we found that when gossipers’ outcomes depended on the targets compared to the no independence condition, they were more likely to share untrustworthy gossip. Specifically, gossip about uncooperative targets was more often false, misrepresenting these targets as cooperative. Counter to H2, we did not find that gossipers were more likely to share untrustworthy gossip when their outcomes depended on receivers compared to the no independence condition, they were not significantly more likely to describe cooperative targets as uncooperative if their outcomes depended on receivers—although this would have been an economically rational decision to increase their outcomes.

## General Discussion

To understand to which extent gossip is helpful to obtain reliable knowledge of people’s social world, it is essential to grasp when, why, and how gossip is trustworthy or untrustworthy (Giardini et al., 2022; Peters & Fonseca, 2020; Wu et al., 2021). To answer this question, we integrated two disparate schools of thought that have studied gossip in different contexts and came to different conclusions about the motives for gossip. We built on theories of interdependence within the gossip triad and reciprocity (Dores Cruz, Thielmann et al., 2021; Giardini & Wittek, 2019; Wu et al., 2021) and argued that contexts involving interdependence between the parties involved in gossip are associated with more self-serving and untrustworthy gossip whereas contexts lacking such interdependence are associated with more prosocial and trustworthy gossip. To contribute to a more comprehensive understanding of relevant social contexts that shape gossip than the previous separate schools of thought on gossip enabled, we investigated gossip’s trustworthiness in different interdependence structures in an online scenario study (Study 1) and a preregistered interactive lab experiment (Study 2).

Generally, and despite the negative connotation of gossip, people gossiped more often than not, and most gossip was true. At the same time, the interdependence structure in which gossip occurred impacted gossip’s trustworthiness: When gossiper’s outcomes were dependent on targets compared to independent from others, gossipers more frequently falsely described an uncooperative target as cooperative in their gossip; also in this context, such self-serving false positive gossip was more likely than false negative gossip describing a cooperative target as uncooperative. This preference for false positive gossip led to higher outcomes for targets and senders at the expense of receivers, as receivers tended to reciprocate the behavior of targets’ behavior that was described in received gossip. Such false gossip made up more than half of the gossip about defecting targets (any false gossip in other conditions made up maximally one-tenth of gossip; in Study 1, this was one-third of gossip, with false gossip in other conditions making up maximally one-fifth of gossip). This shows that people strategically gossip about their allies to rivals when this benefits them (cf. McAndrew et al., 2007). Thus, receivers of gossip rightfully use information about the relationship between gossipers and targets to decide the trustworthiness of gossip (Dores Cruz, Thielmann, et al., 2021; Hess & Hagen, 2006a; Liberman & Shaw, 2020).

We had also expected senders to be more likely to misrepresent cooperative targets as uncooperative if their outcomes depended on the receiver as this would be economically rational to maximize their profits. However, the data from both studies did not support this hypothesis. This contradicts the idea of the homo economicus (E. Fehr & Fischbacher, 2003; Skitka et al., 2008; Yamagishi et al., 2014). It seems like people were averse to damaging cooperative targets, even if this would have been beneficial for themselves. Future research is required to understand people’s perceptions of different lies in gossip.

A possible explanation could be that people preferred to share truthful information about cooperation because mutual cooperation was not the worst possible outcome. This strategy could result in either moderate outcomes (in Study 2: €3 if a receiver cooperated) or high outcomes (€6 if a receiver defected). This strategy further implied that senders did not have to actively play a malicious role to acquire higher benefits because receivers could use that information about cooperation to reciprocate cooperation, or in contrast, to exploit targets’ cooperation. Such behavior has been referred to as ethical free-riding (Gross et al., 2018). This is supported by the finding that people were especially likely to gossip when they were interdependent with receivers and targets cooperated (96.1% in Study 1; 83.6% in Study 2). In contrast, such a strategy was not possible when senders were interdependent with targets. If targets defected, senders could either earn the highest possible outcome or nothing. This could make the incentive for actively lying to manipulate receivers higher in the interdependent with the target condition.

As such, interdependence could affect untrustworthy gossip in different ways. We found that only interdependence with targets was related to more self-serving false gossip compared to no interdependence while there was no such increase for interdependence with receivers. In the latter context, if the receiver does not trust the false negative gossip and does not reciprocate what the target allegedly did, this benefits the collective because mutual cooperation is achieved. This greater possibility of positive outcomes following (even false) gossip might contribute to understanding the prevalence of gossip in everyday life as well as its role in promoting cooperation. Studies have found that most gossip occurs in interdependence structures that align with the interdependence with the receiver condition, showing gossip senders and receivers often have a strong positive relationship (Dores Cruz, Thielmann, et al., 2021; Wittek & Wielers, 1998). Therefore, a large portion of everyday gossip seems to occur in a context that, according to our findings, does not promote proself false gossip and in which true and false gossip could (unintentionally) lead to cooperation.

Another alternative strategy could be to refrain from gossip and let others determine one’s outcomes, representing a different form of ethical free-riding (Gross et al., 2018). We found that the decision to (not) gossip was also influenced by the interdependence structure. First, when senders were interdependent with targets and could benefit from targets exploiting receivers’ cooperation, their overall propensity to gossip did not increase compared to when they could not benefit in the no interdependence condition. Taking a more fine-grained look, solely when targets defected, lying and refraining from gossip were roughly equally frequent and more frequent than true gossip in Study 2 (for an overview of decisions to gossip per condition, see Supplemental Material). This could indicate that observers refrained from gossiping for selfish reasons. This is supported by Study 1 where refraining from gossip about defection in the interdependent with the target condition was more frequent than true and false gossip. It is possible, however, that the absence of real incentives or the strategy method made participants more likely to opt for ethical free-riding rather than engaging in false gossip.

Second, gossip was more likely in the interdependent with the receiver condition (compared to the no interdependence condition), where participants were especially likely to gossip truthfully about cooperation (in Study 1, true gossip about cooperation was also more likely than false gossip or silence). Sharing true gossip about cooperation could have similar benefits and costs to remaining silent. Yet, true gossip about cooperation is morally unobjectionable and provides senders with more control over their outcomes because it can influence receivers’ reactions in way that can increase the likelihood of gossipers achieving their preferred outcome.

Gossip did indeed provide control over receivers’ behavior across both studies, as explorative analyses showed that receivers tended to reciprocate the behavior that was described in gossip. Compared to when no gossip was received, receivers were more likely to cooperate with a target that was described as cooperative and less likely to cooperate with a target described as uncooperative. This indicates that senders can strategically use (false) gossip to get receivers to behave in ways that fit their goals, even when receivers are aware that gossip can be false (Dores Cruz, Van der Lee, & Beersma, 2021; Fonseca & Peters, 2018).

Moreover, the effect of gossip (compared to no gossip) on receivers’ behavior was equally large for gossip about cooperation and defection. Unfortunately, this indicates that receivers responded equally to information that was likely to be true and information that was likely to be false. However, receivers were generally more likely to defect than cooperate in Study 2: Even after receiving gossip about cooperation, the probability of cooperating was approximately 50%. This could indicate that our participants remained cautious or were looking to exploit others for personal gain. Therefore, future empirical work is needed to understand the impact of positive and negative gossip as some previous work indicates negative information weighs stronger than positive information (Baumeister et al., 2001) while others suggest that this is not the case for gossip (Imada et al., 2021).

Our results imply that choosing to engage in gossip or refrain from gossip is a strategic, non-trivial decision that can shape receivers’ behavior in a way that benefits or harms all parties involved. We find that people decide to gossip more often than not. This could indicate that people are motivated to use gossip strategically to shape social situations both positively, protecting receivers and promoting cooperation, and negatively, by benefiting themselves as gossip senders. Moreover, gossip is not only biased by true or false content, but also by whether information gets shared in the first place. We find that people also have strategic reasons not to gossip. Therefore, silence may not be more ethical than gossip, especially as most gossip in our studies was true. This points to the importance of allowing people to decide whether to gossip or refrain from gossip in experimental settings and studying gossip *and* refraining from gossip in the field to fully understand gossip’s trustworthiness.

### Limitations

That said, in both our studies, people could share one observation with one receiver who could not directly interact with them. It is possible that in real life the decision to gossip or not is affected by other (strategic) considerations such as possessing more and/or different information, having a choice of receivers, and whether gossip can be reciprocated (e.g., Beersma et al., 2019; Dores Cruz et al., 2019; Lee & Barnes, 2021; Wu et al., 2016b; Wu et al., 2021).

Furthermore, another limitation of our study designs is that false positive gossip and false negative gossip could lead to different benefits for gossipers in the two interdependence structure conditions that involved interdependence. We, therefore, did not compare the prevalence of false positive and false negative gossip between them but tested differences within conditions or compared each condition involving interdependence with the no interdependence condition. To illustrate, in the interdependent with the target condition, there was an incentive for false positive gossip (gossiping that the target cooperated while they actually defected). In the interdependent with the target condition, there was an incentive for false negative gossip (gossiping that the target defected while they actually cooperated). In Study 2, false positive gossip could increase the likelihood of the receiver cooperating. If the gossiper is interdependent with the target, this means that they gained €6, instead of €0 when both parties would defect. This resulted in a benefit of €6 for the gossiper. Similarly, false negative gossip could increase the likelihood of the receiver defecting. If the gossiper is interdependent with the receiver, this means that they gained €6, instead of €3 when both parties would cooperate. This resulted in a benefit of €3 for the gossiper. The incentive for false negative gossip in the interdependent with the receiver condition was thus smaller than the incentive for false positive gossip in the interdependent with the target condition.

It is important to point out that this difference in incentives is inherent to the structure of both the weak and the standard prisoner’s dilemma. If we assume that the prisoner’s dilemma reflects at least some everyday interactions (Columbus et al., 2021), the potential benefits of engaging in positive and negative false gossip might differ in everyday life as they did in our studies. This means positive and negative false gossip are not comparable because they yield different incentives. Future research could design artificial experimental situations in which these types of false gossip yield equal outcomes to disentangle whether differences in the frequency of positive and negative false gossip are driven by the differences in incentives or by inherent differences in what it means for senders to share false negative gossip versus false positive gossip.

Two other limitations must be considered. First, because in each round, participants interacted with new group members anonymously, there was no impact of gossip behavior on the reputation of senders (similarly in the single-shot design used Study 1). This might have increased participants’ tendency to gossip compared to real-life situations, where people tend to perceive gossiping as negative (Farley, 2011). This could also have made people more likely to gossip for selfish reasons because there were no consequences for spreading untrustworthy information, or conversely, no opportunities to gain benefits from being perceived as a prosocial gossiper (e.g., Feinberg et al., 2012; Giardini & Conte, 2011). However, as this was the same across experimental conditions, it is still possible to draw conclusions about differences in the trustworthiness of gossip between the interdependence structures. Future research could investigate whether gossipers change their behavior when repeatedly gossiping to the same receiver or when their reputation is otherwise at stake.

Second, a potential limitation of Study 2 is the use of a weak prisoner’s dilemma (with equal outcomes for mutual defection and for a cooperator whose counterpart defects). We employed the weak prisoner’s dilemma to increase the prevalence of cooperation by targets to gossip about, as a more even distribution of cooperation and defection prevents a low number of observations for specific types of gossip. In the weak prisoner’s dilemma, defection is only weakly dominant while other properties are similar to the standard prisoner’s dilemma. In line with this, previous work discussed similarities between the weak and standard prisoner’s dilemmas and found more cooperation in the weak prisoner’s dilemma (Burton-Chellew & West, 2012 ; Rapoport, 1988; van den Assem et al., 2012). Compared to the standard prisoner’s dilemma, it is possible that the weak prisoner’s dilemma led gossipers to send more prosocial lies that benefit targets or have a stronger tendency to refrain from gossip about defection because a null outcome for everyone could be seen as immoral or inefficient (Charness & Rabin, 2002; Tappin & Capraro, 2018). Yet, we found no evidence for this; the pattern of false gossip was similar in Study 1 (standard prisoner’s dilemma) and Study 2 (weak prisoner’s dilemma).

## Conclusion

Despite these limitations, our findings contribute to a growing body of research showing that people generally tend to share more true than false information about others, indicating that gossip can be considered trustworthy (Fonseca & Peters, 2018; Giardini et al., 2022; Peters & Fonseca, 2020). This seems to support the prosocial role ascribed to gossip in promoting cooperation (e.g., Feinberg et al., 2012, 2014; Milinski, 2019; Wu et al., 2016b), for which a degree of accuracy is needed for gossip to be successful (D. Fehr & Sutter, 2019; Fonseca & Peters, 2018; Giardini et al., 2022). We add the insight that negative gossip was more often trustworthy than not. This is an indication of the potential value of negative gossip, which is common in our everyday lives (Dores Cruz, Thielmann, et al., 2021; Dores Cruz, Van der Lee, & Beersma, 2021; Robbins & Karan, 2019), to form accurate reputations of others as previous research indicates that negative information is more diagnostic of targets’ cooperativeness and that people are generally more attuned to negative information (e.g., Baumeister et al., 2001; Brambilla et al., 2021).

Moreover, we build on previous findings and theorizing that pointing that the relationships between the parties involved in gossip are key to understanding if gossip is trustworthy (Dores Cruz, Thielmann, et al., 2021; Peters & Fonseca, 2020; Wu et al., 2021). For example, when gossipers are in competition with others or benefit more from one party doing better than another, gossip is more likely to be false. When the interdependence context allows gossipers to benefit themselves via the target’s outcomes, the proportion of false gossip reached levels at which gossip was found to no longer be able to promote cooperation and rather would lead to breakdowns of cooperation (D. Fehr & Sutter, 2019; Fonseca & Peters, 2018; Giardini et al., 2022). This supports findings on proself gossip and further supports that the underlying interdependence structure determines if gossip is used for selfish reasons (e.g., Hess & Hagen, 2021; McAndrew et al., 2007). It is therefore essential that future research considers the interdependence structure (or manipulates it), to interpret findings on the antecedents, content, and consequences of gossip.

In conclusion, our findings inform an integrative perspective on gossip suggesting that gossip is not either trustworthy or untrustworthy. Rather, the context in which gossip occurs shapes its trustworthiness: Gossip is sometimes untrustworthy and self-serving and sometimes it is trustworthy and prosocial. Our results also suggest that, across interdependence contexts, gossip is often more trustworthy and less malicious than its lay reputation suggests (e.g., Dores Cruz, Nieper, et al., 2021) while, concurrently, it might be less trustworthy as a basis of cooperation than lab research suggests (e.g., Feinberg et al., 2012; Sommerfeld et al., 2007). Interdependence increases self-serving motives when gossipers are interdependent with targets. To understand gossip and its functions in our everyday social lives, it is therefore essential for future research to explicitly consider the context in which gossip occurs. Future studies could build on the current work by comparing different interdependence structures characterizing the relationships between the parties involved in gossip, such as negative interdependence and interactions between positive and negative interdependence (Wu et al., 2021).

## Supplemental Material

sj-docx-1-psp-10.1177_01461672231171054 – Supplemental material for Nasty and Noble Notes: Interdependence Structures Drive Self-Serving Gossip Supplemental material, sj-docx-1-psp-10.1177_01461672231171054 for Nasty and Noble Notes: Interdependence Structures Drive Self-Serving Gossip by Terence D. Dores Cruz, Romy van der Lee, Myriam N. Bechtoldt and Bianca Beersma in Personality and Social Psychology Bulletin

## Notes

1. Interdependence refers to the extent to which parties influence each other’s outcomes (focusing on ultimate goals: survival and reproduction, or on proximate goals: how people affect each other; for details see Balliet et al., 2016; Wu et al., 2021).

2. Here, truthful gossip refers to gossip that correctly describes a target as cooperative or uncooperative, and false gossip refers to gossip that misrepresents a cooperative (uncooperative) target as uncooperative (cooperative). It is possible that people share false gossip that exaggerates the degree of (un)cooperativeness of a target (see Peters & Fonseca, 2020). This false gossip serves a similar function as the truthful gossip we examine here.

3. We had preregistered analyses comparing the interdependence conditions; however, as pointed out by an anonymous reviewer, differences in incentives (see general discussion) made these conditions incomparable. Therefore, we did not test these hypotheses.

4. Senders were instructed that they could only send information about the behavior and/or character of targets. Senders were further not allowed to share information about their own or others’ outcomes nor to provide information that could identify them (this was also the case in Study 2).

5. Participants were recruited in two waves from March to April in 2019 and in 2020. Participants in 2020 engaged in slightly more false gossip in the role of sender and decided to defect slightly more often in the role of receiver, see Supplemental Material for details.

6. In the regular prisoner’s dilemma, defection is the dominant strategy to maximize outcomes. In the weak prisoner’s dilemma, defection is a weakly dominant strategy (defection is at least as good as cooperation or better). This makes cooperation a better strategy in the weak prisoner’s dilemma relative to the regular prisoner’s dilemma.

7. Sixteen rounds were excluded from these analyses because participants who indicated that they wanted to gossip failed to write a message within the provided time of 80 seconds. This time limit was set to prevent excessive waiting.
