# Supplementary material for: Nasty and Noble Notes: Interdependence Structures Drive Self-Serving Gossip
Source: Pers Soc Psychol Bull. 2023 May 25;50(11):1596–612. doi: 10.1177/01461672231171054 (PMC11492547; doi:10.1177/01461672231171054)
Supplement: sj-docx-1-psp-10.1177_01461672231171054 – Supplemental material for Nasty and Noble Notes: Interdependence Structures Drive Self-Serving Gossip [file sj-docx-1-psp-10.1177_01461672231171054.docx]

**Supplemental Material**

**Nasty and Noble Notes: Interdependence Structure Drives Self-serving Gossip**

[Study 1 2](#_Toc134478808)

[Supplemental Methods Study 1 2](#_Toc134478809)

[Open Text Messages Gossip 3](#_Toc134478810)

[Comprehension Questions 3](#_Toc134478811)

[Perceptions of Gossip and the Interdependence Structure. 3](#_Toc134478812)

[Statistical Analyses Study 1 4](#_Toc134478813)

[Supplemental Results Study 1 5](#_Toc134478814)

[Overview of Sender Gossip and Refraining From Gossip 5](#_Toc134478815)

[Comparisons of Self-Serving False Gossip versus Not Self-Serving False Gossip Controlling for Age and Gender. 9](#_Toc134478816)

[Perceptions of Gossip and the Interdependence Structure 12](#_Toc134478817)

[Receiver Cooperation After Receiving Gossip (or Not) 14](#_Toc134478818)

[Study 2 17](#_Toc134478819)

[Supplemental Methods Study 2 17](#_Toc134478820)

[Open Text Messages Gossip 17](#_Toc134478821)

[Comprehension Questions 17](#_Toc134478822)

[Statistical Analyses Study 2 17](#_Toc134478823)

[Supplemental Results Study 2 19](#_Toc134478824)

[Overview of Sender Gossip and Refraining From Gossip 19](#_Toc134478825)

[Complete Models With Controls for Gossip Veracity Study 2 22](#_Toc134478826)

[Complete Models With Controls for The Decision to Gossip Study 2 29](#_Toc134478827)

[Complete Models with Controls for Receiver Cooperation Study 2 34](#_Toc134478828)

[Robustness Check Using Generalized Estimating Equations for Study 2 Hypotheses 37](#_Toc134478829)

[Pre-registered Test of Interaction With Social Value Orientation (SVO) 41](#_Toc134478830)

[References 42](#_Toc134478831)

# Study 1

## Supplemental Methods Study 1

For an overview of the procedure, see Figure S1. For the outcomes of the prisoner’s dilemma, see Table S1.

**Figure S1.**

*Overview of Study 1 design.*

**

**Table S1.**

*Outcomes in the adapted sequential prisoner’s dilemma game in Study 1.*

|  |  | Target | |
| --- | --- | --- | --- |
|  |  | Cooperate | Defect |
| Receiver | Cooperate | Target: $50  Receiver: $50 | Target: $100  Receiver: $0 |
|  | Defect | Target: $0  Receiver: $100 | Target: $0  Receiver: $0 |

*Note.* The table included the observer with the same amount as the target or receiver in the corresponding interdependence structure condition.

### Open Text Messages Gossip

If participants decided to send gossip to the receiver, they also had to type a free text message (“Please write the text message about the target that you want to share with the receiver”). This message was communicated to the receiver along with the forced-choice gossip. We did not further analyze these messages; they can be found in the data file on https://osf.io/dkjnh.

### Comprehension Questions

We asked participants to imagine being the sender and deciding to gossip or not. After the random assignment to one of two interdependence structure conditions, participants answered four comprehension questions about this (e.g., “If the target chooses Option 2 and the receiver chooses Option 1, how much do you (the sender) earn?”; participants could only continue when they answered correctly with 100).

### Perceptions of Gossip and the Interdependence Structure.

After making their decisions as the sender, participants answered questions about how their gossip could impact themselves, the receiver, and the target. All items were answered on Likert-scale from 1 (*completely disagree)* to 7 (*completely agree)*. Participants rated to what extent gossip about the target could benefit the target/receiver/themselves, protect the target/receiver/themselves, and maximize the earnings of the target/receiver/themselves. Items concerning the target were combined into a single scale (α = .89; *M* = 3.99, *SD* = 1.81). Items concerning the receiver were combined into a single scale (α = .89; *M* = 5.20, *SD* = 1.59). Items for the participant’s own benefit were combined into a single scale (α = .87; *M* = 5.79, *SD* = 1.32). A final question asked participants to what extent communication could influence the target to act in their favor (*M* = 3.03, *SD* = 2.09).

### Statistical Analyses Study 1

First, to analyze gossip veracity (0 = true, 1 = false) and sender decision to gossip (0 = true, 1 = false), we used generalized (binomial) mixed models with the interdependence structure condition (0= interdependence with the receiver, 2 = interdependence with the target), and target decision (0 = cooperate, 1 = defect) and the interaction between interdependence structure condition and target decision as independent variables. All models included random intercepts for participants (we report results without control variables, results did not differ when controlling for age and gender)^[[1]](#footnote-1)^.

Second, to analyze the receiver decision to cooperate (0 = defect, 1 = cooperate), we used a generalized (binomial) mixed model with the gossip event (0 = gossip about cooperation, 1 = gossip about defection, 3 = no gossip) as the independent variable.

Analyses were performed in R (Version 4.1.2, R Core Team, 2021). All tests were two-sided. In R, mixed-effects models were fitted with REML estimation using the package ‘lme4’ (Version 1.1-27.1, Bates et al., 2015) and ANOVA significance tests on these models were performed using Wald χ^2^ tests using the package 'car' (Version 3.0-11, Fox & Weisberg, 2019). Post-hoc pairwise comparison analyses with a Bonferroni correction (for the decision to gossip and the receiver cooperation) were performed using the package 'emmeans' (Version 1.6.2-1; Lenth, 2021).

## Supplemental Results Study 1

### Overview of Sender Gossip and Refraining From Gossip

Table S2 presents an overview of the frequency of gossip and refraining from gossip across interdependence structure conditions and target decisions.

We compared within conditions whether engaging in gossip was more likely when the target defected or cooperated. There was a significant interaction between the interdependence structure conditions and target decision (Wald χ2(1) = 5.75, *p* = .017; controlling for age and gender: Wald χ2(1) = 5.96, *p* = .015). In the interdependent with the target condition, gossip was less likely when the target defected compared to when the target cooperated (*OR* = 0.30, *SE* = 0.08, *z* = -4.25, *p* < .001; controlling for age and gender: *OR* = 0.29, *SE* = 0.08, *z* = -4.29, *p* < .001). In the interdependent with the receiver condition, gossip was not significantly more or less likely when the target defected compared to when the target cooperated (*OR* = 1.26, *SE* = 0.40, *z* = 0.75, *p* = .450; controlling for age and gender: *OR* = 1.26, *SE* = 0.39, *z* = 0.76, *p* = .447). Below in Table S3, we present an overview of post-hoc comparisons with a Bonferroni correction that compare the probability of gossip across all levels of the interdependence conditions and target decisions.

We did also find a significant main effect of the interdependence structure condition, showing that gossip was more likely when senders were interdependent with the receiver compared to when senders were interdependent with the target (*OR* = 5.02, *SE* = 1.51, *z* = 5.37, *p* < .002; controlling for age and gender: *OR* = 5.67, *SE* = 1.78, *z* = -5.54, *p* < .001; Please interpret this comparison with great caution because these conditions have different incentives for (false) gossip that could make the conditions incomparable as discussed in the main manuscript). There was no significant main effect of the target decision (Wald χ2(1) = 0.57, *p* = .450; controlling for age and gender: Wald χ2(1) = 0.57, *p* = .447). In the model controlling for gender and age, there was also a significant effect of gender such that women were less likely to gossip than men (*OR* = 0.51, *SE* = 0.14, *z* = -2.41, *p* = .016).

**Table S2.**

*Frequencies of gossip and refraining from gossip across interdependence structure conditions and target decisions. Percentages represent the percentage of a behavior within a condition for a decision of the target (Study 1).*

|  | Interdependence structure condition | | | | | | | | |
| --- | --- | --- | --- | --- | --- | --- | --- | --- | --- |
|  | Interdependent with the receiver | | | Interdependent with the target | | | Overall | |  |
| Target decision | True gossip | False gossip | Refrain from gossip | True  gossip | False gossip | Refrain from gossip | True  gossip | False  gossip | Refrain from gossip |
| Cooperate | 122 (75.8%) | 5 (3.1%) | 34 (21.1%) | 75 (68.1%) | 20 (12.8%) | 61 (39.1%) | 197 (62.1%) | 25 (7.9%) | 95 (30%) |
| Defect | 106 (66.2%) | 15 (9.4%) | 39 (24.4%) | 45 (27.4%) | 23 (14%) | 96 (58.5%) | 151 (46.6%) | 38 (11.7%) | 135 (41.7%) |
| Overall | 228 (71%) | 20 (6.2%) | 73 (22.7%) | 120 (37.5%) | 43 (13.4%) | 157 (49.1%) | 348 (54.3%) | 63 (9.8%) | 230 (35.9%) |

**Table S3.**

*All post-hoc comparisons for the decision to gossip with a Bonferroni correction (Study 1).*

|  |  |  | Model without controls | | | Model controlling for gender and age | | |
| --- | --- | --- | --- | --- | --- | --- | --- | --- |
| Contrast decision to gossip |  |  | *OR* (*SE*) | *Z* | *p* | *OR*  (*SE*) | *Z* | *p* |
| Interdependent w/ receiver & target cooperated | - | Interdependent w/ target & target cooperated | 3.06 (1.08) | 3.18 | .009 | 3.40 (1.23) | 3.73 | .005 |
| Interdependent w/ receiver & target cooperated | - | Interdependent w/ receiver & target defected | 1.26 (0.38) | 0.75 | > .999 | 1.26 (0.39) | 0.76 | > .999 |
| Interdependent w/ receiver & target cooperated | - | Interdependent w/ target & target defected | 10.37 (4.05) | 5.99 | < .001 | 11.94 (4.83) | 6.12 | < .001 |
| Interdependent w/ target & target cooperated | - | Interdependent w/ receiver & target defected | 0.41 (0.14) | -2.60 | .056 | 0.37 (0.13) | -2.81 | .030 |
| Interdependent w/ target & target cooperated | - | Interdependent w/ target & target defected | 3.39 (0.98) | 4.25 | < .001 | 3.52 (1.03) | 4.29 | < .001 |
| Interdependent w/ receiver & target defected | - | Interdependent w/ target & target defected | 8.25 (3.11) | 5.59 | < .001 | 9.46 (3.70) | 5.75 | < .001 |

### Comparisons of Self-Serving False Gossip versus Not Self-Serving False Gossip Controlling for Age and Gender.

Without controlling for age and gender, in the interdependent with the target condition, self-serving false positive gossip (i.e., describing a defecting target as having cooperated) was not more likely than false negative gossip that was not self-serving (i.e., describing a cooperating target as having defected; *OR* = 2.39, *SE* = 1.11, *z* = 1.88, *p* = .060). When adding age and gender as control variables, self-serving false positive gossip was also not more likely than false negative gossip that was not self-serving (*OR* = 2.28, *SE* = 1.03, *z* = 1.84, *p* = .067).

Without controlling for age and gender, in the interdependent with the receiver condition, self-serving false negative gossip (i.e., describing a cooperating target as having defected) was significantly less likely than false positive gossip that was not self-serving (i.e., describing a defecting target as having cooperated; *OR* = 0.25, *SE* = 0.15, *z* = -2.38, *p* = .017). When adding age and gender as control variables, self-serving false positive gossip was also less likely than false negative gossip that was not self-serving (*OR* = 0.24, *SE* = 0.14, *z* = -2.45, *p* = .014).

Below in Table S4, we present an overview of post-hoc comparisons with a Bonferroni correction that compare the probability of false gossip across all levels of the interdependence conditions and target decisions. It must be noted that there was no significant interaction between the interdependence structure conditions and target decision (Wald χ2(1) = 0.54, *p* = .460; controlling for age and gender: Wald χ2(1) = 0.72, *p* = .397).

We did find a significant main effect of the interdependence structure condition, showing that false gossip was less likely when senders were interdependent with the receiver compared to when senders were interdependent with the target (*OR* = 0.15, *SE* = 0.07, *z* = -3.91, *p* < .001; controlling for age and gender: *OR* = 0.17, *SE* = 0.08, *z* = -3.91, *p* < .001; please interpret this comparison with great caution because these conditions have different incentives for false gossip that could make the conditions incomparable as discussed in the main manuscript). There was also a significant main effect of the target decision, showing that false gossip was less likely when the target had cooperated compared to when the target had defected (*OR* = 0.32, *SE* = 0.13, *z* = -2.90, *p* = .004; controlling for age and gender: *OR* = 0.33, *SE* = 0.12, *z* = -2.95, *p* = .003). In the model controlling for gender and age, there was also a significant effect of age, such that older participants were less likely to gossip falsely (*Est*. (*SE*) = -0.04 (0.02), *z* = -2.10, *p* = .036).

**Table S4.**

*All post-hoc comparisons for gossip veracity with a Bonferroni correction (Study 1).*

|  |  |  | Model without controls | | | Model controlling for gender and age | | |
| --- | --- | --- | --- | --- | --- | --- | --- | --- |
| Contrast gossip veracity |  |  | *OR* (*SE*) | *Z* | *p* | *OR*  (*SE*) | *Z* | *p* |
| Interdependent w/ receiver & target cooperated | - | Interdependent w/ target & target cooperated | 0.11 (0.07) | -3.33 | .005 | 0.12 (0.08) | -3.34 | .001 |
| Interdependent w/ receiver & target cooperated | - | Interdependent w/ receiver & target defected | 0.25 (0.15) | -2.38 | .105 | 0.24 (0.14) | -2.45 | .085 |
| Interdependent w/ receiver & target cooperated | - | Interdependent w/ target & target defected | 0.05 (0.04) | -4.09 | < .001 | 0.05 (0.04) | -4.14 | < .001 |
| Interdependent w/ target & target cooperated | - | Interdependent w/ receiver & target defected | 2.16 (1.02) | 1.62 | .632 | 1.96 (0.91) | 1.46 | .872 |
| Interdependent w/ target & target cooperated | - | Interdependent w/ target & target defected | 0.42 (0.19) | -1.88 | .357 | 0.44 (0.20) | -1.84 | .399 |
| Interdependent w/ receiver & target defected | - | Interdependent w/ target & target defected | 0.20 (0.11) | -2.98 | .017 | 0.22 (0.12) | -2.84 | .027 |

*Note*. Due to different incentives for false gossip (see main text), we advise caution in interpreting differences between the conditions.

### Perceptions of Gossip and the Interdependence Structure

Table S5 and Figure S2 presents an overview of how senders thought gossip could affect them and other parties in the interdependence structure conditions. In both conditions, senders reported that gossip could benefit themselves and other parties, but they also reported that their gossip could positively impact the party that they were interdependent with, which would also impact their own outcomes (while gossip could be equally impactful on each party in each condition). In both supporting items that captured gossip benefitted the senders were supported.

**Table S5.**

*Means and standard errors for support for statements on how gossip could affect different parties across interdependence structure conditions (Study 1).*

|  | Interdependence Structure Condition | | | |
| --- | --- | --- | --- | --- |
|  | Interdependent with the receiver | | Interdependent with the target | |
|  | *M* | *SD* | *M* | *SD* |
| Gossip could be good for the target | 3.31 | 1.69 | 4.67 | 1.68 |
| Gossip could be good for the receiver | 5.76 | 1.24 | 4.63 | 1.70 |
| Gossip could be good for the sender themselves | 6.04 | 1.07 | 5.54 | 1.48 |
| Gossip could influence the target to act in the sender’s favor | 2.66 | 1.89 | 3.40 | 2.21 |

**Figure S2.**

*Perceived benefits of gossip per interdependence structure condition in Study 1.*

*Note.* Dots represent individual data points. The box limits show the 25th percentile, the median, and the 75th percentile, respectively. The whiskers extend to 1.5 times the interquartile range. Diamonds indicate the mean with the error bars representing a 95% confidence interval. Note we did not statistically compare between the interdependence conditions due to different incentives for false gossip (see main text), we advise caution therefore in interpreting differences between the conditions.

### Receiver Cooperation After Receiving Gossip (or Not)

We investigated how receivers' decisions after receiving gossip to gain insights into whether gossip worked for senders to achieve their goals. We found that receivers' decisions differed based on whether they would receive gossip about cooperation, about defection, or no gossip, see Figure S3 (Wald χ^2^(2) = 137.46, *p* < .001, i.e., effect of the decision of the gossip sender). Specifically, pairwise comparisons showed that receivers who would see gossip describing a target as cooperative were more likely to cooperate (probability = 71.2%, *SE* = 0.03) compared to when they would receive gossip describing a target as defecting (probability = 21.6%, *SE* = 0.02; *OR* = 8.98, *SE* = 1.81, *z* = 10.85, *p* < .001) but not compared to when they would not receive gossip (probability = 68.2% *SE* = 0.03; *OR* = 1.16, *SE* = 0.20, *z* = 0.85, *p* > .999). Moreover, receivers that saw gossip that described a target as uncooperative were less likely to cooperate compared to when they did not receive gossip (*OR* = 0.13, *SE* = 0.03, *z* = -10.34, *p* < .001). Therefore, senders' gossip would be successful in getting receivers to act in line with the content of their gossip for descriptions of the target as cooperative and uncooperative. When receiving no gossip, it seems receivers acted similarly to when gossip about cooperation was received. In sum, gossip could successfully impact receiver cooperation as receivers were more likely to cooperate when gossip described targets as having cooperated compared to defected.

Results were similar when controlling for age and gender. Receivers who would see gossip describing a target as cooperative were more likely to cooperate (probability = 71.5%, *SE* = 0.03) compared to when they would receive gossip describing a target as defecting (probability = 22.1%, *SE* = 0.02; *OR* = 8.86, *SE* = 1.79, *z* = 10.78, *p* < .001) but not compared to when they would not receive gossip (probability = 69.1% *SE* = 0.03; *OR* = 1.13, *SE* = 0.19, *z* = 0.69, *p* > .999). Moreover, receivers that saw gossip that described a target as uncooperative were less likely to cooperate compared to when they did not receive gossip (*OR* = 0.13, *SE* = 0.03, *z* = -10.37, *p* < .001).

There was a significant effect of gender such that women were more likely to cooperate than men (*OR* = 1.38, *SE* = 0.23, *z* = 1.98, *p* = .048). There was further a significant effect of age, such that older participants were less likely to cooperate (*est*. (*se*) = -0.02 (0.01), *z* = -2.39, *p* = .017).

**Figure S3.**

*Probability of cooperation by receivers when gossip about cooperation was received, gossip about defection was received, and when no gossip was received in Study 1. Brackets indicate significant differences.*

*Note*. The error bars indicate a 95% confidence interval. The dots indicate individual observations of cooperation and defection.

# Study 2

## Supplemental Methods Study 2

### Open Text Messages Gossip

If participants decided to send gossip to the receiver, they also had to type a free text message (“Please write the text message about the target that you want to share with the receiver”; minimum of 15 characters). This message was communicated to the receiver along with the forced-choice gossip. We did not further analyze these messages; they can be found in the data file on https://osf.io/af4rc.

### Comprehension Questions

We asked participants plenary comprehension questions after receiving general instructions but before being assigned their role (e.g., “If the target chooses Option 2 and the receiver chooses Option 1, how much does the sender/receiver/target earn?”; If one participant answered incorrectly, the experiment would explain again before continuing.

### Statistical Analyses Study 2

To analyze gossip veracity (0 = true, 1 = false) and sender decision to gossip (0 = no, 1 = yes), we used generalized (binomial) mixed models with the interdependence structure condition (0 [reference] = no interdependence, 1= interdependent with the target, 2 = interdependent with the receiver), and target decision (0 = defect, 1 = cooperate) and the interaction between these variables as independent variables. We used contrast analyses to test H1 (when targets defected: interdependence with the target condition vs no interdependence condition) and H2 (when targets cooperated: interdependence with the receiver condition vs no interdependence condition). Additionally, we tested a contrast between false positive (interdependence with the target condition when the target defected) and false negative gossip (interdependence with the receiver condition when the target cooperated) to explore differences in the frequency of types of proself false gossip. Moreover, we also ran post-hoc tests comparing all conditions and target decisions with each other with a Bonferroni correction. We further used a generalized (binomial) mixed model with gossip content (0 [reference] = no gossip received, 1= gossip about cooperation, 2 = gossip about defection] as the independent variable, using pairwise comparisons with a Bonferroni correction. Additional models for all analyses control for data collection wave, round number, age, and gender).

To test the robustness of our results, we repeated our hypothesis tests using Generalized Estimating Equations. To do this, we used to run binary logistic regression models in which we nested gossip within rounds and subjects. The model included gossip veracity, the interdependence structure condition, target decision, and their two-way and three-way interactions as independent variables. The dependent variable was whether gossip occurred. Further models controlled for the data collection wave, age, and gender.

Analyses were performed in R (Version 4.1.2, R Core Team, 2021). All tests were two-sided. In R, mixed-effects models were fitted with REML estimation using the package ‘lme4’ (Version 1.1-27.1, Bates et al., 2015). Generalized Estimating Equations were to fit binary logistic regression models in which we nested gossip within rounds and subjects using the R package “geepack” (Halekoh et al., 2006). Type III ANOVA significance tests on these models were performed using Wald χ^2^ tests using the package 'car' (Version 3.0-11, Fox & Weisberg, 2019). Contrast analyses and pairwise comparisons using Bonferroni corrections (for the decision to gossip and the receiver cooperation) were performed using the package 'emmeans' (Version 1.6.2-1; Lenth, 2021).

Data were plotted using the packages ‘ggplot2’ (Wickham, 2016), ‘effects’ (Fox & Weisberg, 2019), ‘cowplot’ (Wilke, 2020), and ‘ggsignif’ (Constantin & Patil, 2021).

## Supplemental Results Study 2

### Overview of Sender Gossip and Refraining From Gossip

Table S6 and Figure S4 present an overview of the frequency of gossip and refraining from gossip across interdependence structure conditions and target decisions. Comparing this to table S2 shows that Study 1 also did show that false gossip exhibited a similar pattern as Study 2. On the one hand, false gossip was most likely when senders were interdependent with targets that defected. On the other hand, false gossip was proportionally rare when targets cooperated and when senders were interdependent with receivers. This indicates that untrustworthy gossip occurred particularly when senders could benefit by misrepresenting a defector as a cooperator (false positive gossip) while misrepresenting a cooperator as a defector (false negative gossip) occurred rarely. Descriptively, across both studies, gossip with positive content was more likely to be untrustworthy than gossip with negative content.

**Table S6.**

*Frequencies of gossip and refraining from gossip across interdependence structure conditions and target decisions. Percentages represent the percentage of a behavior within a condition for a decision of the target (Study 2).*

|  |  |  |  | | Interdependence structure condition | | | | | | | |  |
| --- | --- | --- | --- | --- | --- | --- | --- | --- | --- | --- | --- | --- | --- |
|  | No interdependence | | | Interdependent with the target | | | | Interdependent with the receiver | | | Overall | |  |
| Target decision | True gossip | False gossip | Refrain from gossip | True gossip | | False gossip | Refrain from gossip | True  gossip | False gossip | Refrain from gossip | True  gossip | False  gossip | Refrain from gossip |
| Cooperate | 257 (56.1%) | 32 (6.99%) | 169 (36.9%) | 232 (51.7%) | | 48 (10.7%) | 169 (37.6%) | 328 (74.6%) | 40 (9.1%) | 72 (16.4%) | 817 (60.6%) | 120 (8.91%) | 410 (30.4%) |
| Defect | 223 (41.2%) | 86 (15.9%) | 232 (42.9%) | 139 (25.1%) | | 203 (36.7%) | 211 (38.2%) | 341 (60.1%) | 65 (11.5%) | 161 (28.4%) | 703 (42.3%) | 354 (21.3%) | 604 (36.4%) |
| Overall | 480 (48.0%) | 118 (11.8%) | 401 (40.1%) | 371 (37.0%) | | 251 (25.0%) | 380 (37.9%) | 669 (66.4%) | 105 (10.4%) | 233 (23.1%) | 1520 (50.5%) | 474 (15.8%) | 1014 (33.7%) |

**Figure S4.**

*Frequencies of gossip and refraining from gossip across interdependence structure conditions and target decisions. Percentages represent the percentage of a behavior per condition within gossip or refraining from gossip (Study 2).*

*Note*. False positive gossip indicates misrepresenting a cooperative target as uncooperative, false negative gossip indicates misrepresenting an uncooperative target as cooperative, true positive gossip indicates representing a cooperative target as cooperative, and true negative gossip indicates representing an uncooperative target as uncooperative.

### Complete Models With Controls for Gossip Veracity Study 2

Below we present an overview of models predicting whether gossip was true or false (gossip veracity) using the interdependence structure condition, the decision of the target to cooperate or defect, and their interaction. Further models controlled for the round number, the data collection, age, and gender. Interpretation of significant main effects are presented below (see Table S7), the interpretation of the significant interaction can be found in Table S8 and Table S9.

All three models showed a significant main effect of the condition, post-hoc comparisons with a Bonferroni correction showed that gossip was more likely to be false in the interdependent with the target condition compared to the no interdependence condition (model 1 *OR* = 3.00, *SE* = 0.49, *z* = 6.76, *p* < .001; model 2 *OR* = 3.05, *SE* = 0.50, *z* = 6.82, *p* < .001; model 3 *OR* = 3.06, *SE* = 0.50, *z* = 6.84, *p* < .001) and the interdependence with the receiver condition (model 1 *OR* = 4.57, *SE* = 0.03, *z* = 9.62, *p* < .001; model 2 *OR* = 4.57, *SE* = 0.03, *z* = 9.59, *p* < .001; model 3 *OR* = 4.55, *SE* = 0.03, *z* = 9.58, *p* < .001; please interpret this comparison with great caution because these conditions have different incentives for false gossip that could make the conditions incomparable as discussed in the main manuscript). Gossip was also more likely to be false in the no interdependence condition compared to the interdependent with the receiver condition (model 1 *OR* = 1.52, *SE =* 0.11, *z* = 2.55, *p* = .032; model 2 *OR* = 1.49, *SE* = 0.11, *z* = 2.41, *p* = .048; yet model 3 showed no significant difference *OR* = 1.49, *SE* = 0.25, *z* = 2.38, *p* = .053).

All three models also showed a significant main effect of the target decision, post-hoc comparisons showed that gossip was more likely to be false in the interdependent when the target defected compared to cooperated (model 1 *OR* = 3.72, *SE* = 0.04, *z* = -9.54, *p* < .001; model 2 *OR* = 3.82, *SE* = 0.04, *z =* 9.68, *p* < .001; model 3 *OR* = 3.85, *SE* = 0.04, *z* = 9.72, *p* < .001).

Models 2 and 3 showed a significant effect of the data collection wave, gossip less more likely to be false in the first data collection wave in 2020 compared to the second data collection wave in 2019 (model 2 *OR* = 0.60, *SE* = 0.13, *z* = 2.30, *p* = .021; model 3 *OR* = 0.61, *SE* = 0.13, *z* = 2.26, *p* = .024).

Model 3 showed a significant effect of age, older observers were less likely to share false gossip (*Est.* = -0.12, *SE* = 0.05, *z* = 2.59, *p* = .009).

As in Study 1, we also compared within conditions whether false gossip that could benefit senders was more likely than false gossip that could not benefit senders. In the interdependent with the target condition, false positive gossip that could benefit senders was significantly more than false negative gossip that could not benefit senders (Model 1 *OR* = 10.05, *SE* = 2.24, *z* = 10.35, *p* < .001; Model 2 *OR* = 10.56, *SE* = 2.37, *z* = 10.50, *p* < .001; Model 3 *OR* = 10.46, *SE* = 2.35, *z* = 10.46, *p* < .001). In the interdependent with the receiver condition, false negative gossip that could benefit senders was (unexpectedly) significantly less likely than false positive gossip that could not benefit senders (Model 1 *OR* = 0.62, *SE* = 0.14, *z* = -2.10, *p* = .036; Model 2 *OR* = 0.60, *SE* = 0.14, *z* = -2.21, *p* = .027; Model 3 *OR* = 0.59, *SE* = 0.14, *z* = -2.27, *p* = .023). Additionally, in the no interdependence condition where neither type of false gossip could benefit senders, false positive gossip was significantly more likely false negative gossip (Model 1 *OR* = 3.15, *SE* = 0.77, *z* = 4.71, *p* < .001; Model 2 *OR* = 3.16, *SE* = 0.77, *z* = 4.72, *p* < .001; Model 3 *OR* = 3.20, *SE* = 0.78, *z* = 4.77, *p* < .001).

**Table S7.**

*Models predicting gossip using interdependence structure condition, the target decision of the target, and their interaction. Further models controlled for the round number, the data collection, age, and gender as independent variables (Study 2).*

|  | Model 1 | | Model 2 | | Model 3 | |
| --- | --- | --- | --- | --- | --- | --- |
| Predictor of gossip veracity | Wald χ^2^ | *p* | Wald χ^2^ | *p* | Wald χ^2^ | *p* |
| Condition | 7.19 | .027 | 6.87 | .032 | 7.14 | .028 |
| Target decision | 4.39 | .036 | 4.90 | .027 | 5.17 | .023 |
| Condition X Target decision | 34.45 | < .001 | 35.37 | < .001 | 34.44 | <. 001 |
| Round number |  |  | 2.94 | .087 | 3.11 | .078 |
| Data collection wave |  |  | 5.30 | .021 | 5.09 | .024 |
| Age |  |  |  |  | 6.73 | .009 |
| Gender |  |  |  |  | 0.80 | .372 |

*Note*. *df* = 2 for condition and condition X target decision, all other variables *df* = 1. Gossip veracity coded as 0 = true, 1 = false.

**Table S8.**

*Contrasts testing hypothesis 1 (when a target defects, false gossip is more likely in the interdependent with the target condition compared to the no interdependence condition), hypothesis 2 (when a target cooperates, false gossip is more likely in the interdependent with the receiver condition compared to the no interdependence condition; Study 2).*

|  |  |  | Model 1 | | | Model 2 | | | Model 3 | | |
| --- | --- | --- | --- | --- | --- | --- | --- | --- | --- | --- | --- |
| Contrast gossip veracity |  |  | *OR* (*SE*) | *Z* | *p* | *OR* (*SE*) | *Z* | *p* | *OR* (*SE*) | *Z* | *p* |
| Interdependent w/ target & target defected | - | No interdependence & target defected | 5.36 (1.05) | 8.61 | < .001 | 5.58 (1.10) | 8.75 | < .001 | 5.54 (1.09) | 8.72 | < .001 |
| Interdependent w/ receiver & target cooperated | - | No interdependence & target cooperated | 0.91 (0.24) | -0.34 | .732 | 0.92 (0.24) | -0.31 | .758 | 0.93 (0.25) | -0.29 | .770 |

*Note*. Gossip veracity coded as 0 = true, 1 = false.

**Table S9.**

*All post-hoc comparisons for gossip veracity with a Bonferroni correction. Significant differences are in bold (Study 2).*

|  |  |  | Model 1 | | | Model 2 | | | Model 3 | | |
| --- | --- | --- | --- | --- | --- | --- | --- | --- | --- | --- | --- |
| Contrast decision to gossip |  |  | *OR* (*SE*) | *Z* | *p* | *OR* (*SE*) | *Z* | *p* | *OR* (*SE*) | *Z* | *p* |
| No interdependence & target cooperated | - | Interdependent w/ target & target cooperated | 0.60 (0.16) | -2.00 | .691 | 0.60 (0.16) | -1.96 | .743 | 0.59 (0.15) | -2.02 | .654 |
| No interdependence & target cooperated | - | Interdependent w/ receiver & target cooperated | 1.09 (0.29) | 0.34 | > .999 | 1.09 (0.29) | 0.31 | > .999 | 1.08 (0.29) | 0.29 | > .999 |
| **No interdependence & target cooperated** | **-** | **No interdependence & target defected** | **0.32 (0.08)** | **-4.71** | **< .001** | **0.32 (0.08)** | **-4.72** | **< .001** | **0.31 (0.08)** | **-4.77** | **< .001** |
| **No interdependence & target cooperated** | **-** | **Interdependent w/ target & target defected** | **0.06 (0.02)** | **-11.59** | **< .001** | **0.06 (0.01)** | **-11.69** | **< .001** | **0.06 (0.01)** | **-11.70** | **< .001** |
| No interdependence & target cooperated | - | Interdependent w/ receiver & target defected | 0.68 (0.17) | -1.60 | > .999 | 0.65 (0.16) | -1.74 | > .999 | 0.64 (0.16) | -1.81 | > .999 |
| *Interdependent w/ target & target cooperated | - | Interdependent w/ receiver & target cooperated | 1.84 (0.45) | 2.50 | 0.186 | 1.81 (0.44) | 2.43 | .224 | 1.83 (0.45) | 2.48 | .200 |
| Interdependent w/ target & target cooperated | - | No interdependence & target defected | 0.53 (0.12) | -2.82 | 0.072 | 0.53 (0.12) | -2.86 | .064 | 0.53 (0.12) | -2.85 | .065 |
| **Interdependent w/ target & target cooperated** | **-** | **Interdependent w/ target & target defected** | **0.10 (0.02)** | **-10.35** | **< .001** | **0.09 (0.02)** | **-10.50** | **< .001** | **0.10 (0.02)** | **-10.46** | **< .001** |
| *Interdependent w/ target & target cooperated | - | Interdependent w/ receiver & target defected | 1.13 (0.26) | 0.56 | > .999 | 1.09 (0.25) | 0.36 | > .999 | 1.08 (0.25) | 0.34 | > .999 |
| **Interdependent w/ receiver & target cooperated** | **-** | **No interdependence & target defected** | **0.29 (0.07)** | **-5.45** | **< .001** | **0.29 (0.07)** | **-5.42** | **< .001** | **0.29 (0.07)** | **-5.45** | **< .001** |
| ***Interdependent w/ receiver & target cooperated** | **-** | **Interdependent w/ target & target defected** | **0.05 (0.01)** | **-12.81** | **< .001** | **0.05 (0.01)** | **-12.91** | **< .001** | **0.05 (0.01)** | **-12.92** | **< .001** |
| Interdependent w/ receiver & target cooperated | - | Interdependent w/ receiver & target defected | 0.62 (0.14) | -2.10 | 0.543 | 0.60 (0.14) | -2.21 | .403 | 0.59 (0.14) | -2.27 | .345 |
| **No interdependence & target defected** | **-** | **Interdependent w/ target & target defected** | **0.19 (0.04)** | **-8.61** | **< .001** | **0.18 (0.04)** | **-8.75** | **< .001** | **0.18 (0.04)** | **-8.72** | **< .001** |
| **No interdependence & target defected** | **-** | **Interdependent w/ receiver & target defected** | **2.12 (0.43)** | **3.75** | **.003** | **2.06 (0.42)** | **3.56** | **.006** | **2.04 (0.41)** | **3.53** | **.006** |
| ***Interdependent w/ target & target defected** | **-** | **Interdependent w/ receiver & target defected** | **11.39 (2.30)** | **12.08** | **< .001** | **11.47 (2.31)** | **12.11** | **< .001** | **11.31 (2.28)** | **12.05** | **< .001** |

*Note*. Please interpret comparison marked * with great caution because these conditions have different incentives for false gossip that could make the conditions incomparable as discussed in the main manuscript.

### Complete Models With Controls for The Decision to Gossip Study 2

Below we present an overview of models predicting whether observers decided to gossip or refrain from gossip (decision to gossip) using the interdependence structure condition, the decision of the target to cooperate or defect, and their interaction. Further models controlled for the round number, the data collection, age, and gender. Interpretation of significant main effects are presented below (see Table S10), the interpretation of the significant interaction can be found in Table S11.

All three models showed a significant main effect of the condition, post-hoc comparisons with a Bonferroni correction showed that gossip was more likely in the interdependent with the receiver condition compared to the no interdependence condition (model 1 *OR* = 2.81, *SE* = 0.32, *z* = 9.17, *p* < .001; model 2 *OR* = 2.90, *SE* = 0.33, *z* = 9.33, *p* < .001; model 3 *OR* = 2.90, *SE* = 0.04, *z* = 9.32, *p* < .001) and the interdependence with the target condition (model 1 *OR* = 2.54, *SE* = 0.29, *z* = 8.26, *p* < .001; model 2 *OR* = 2.66, *SE* = 0.30, *z* = 8.60, *p* < .001; model 3 *OR* = 2.66, *SE* = 0.30, *z* = 8.60, *p* < .001; please interpret this comparison with great caution because these conditions have different incentives for false gossip that could make the conditions incomparable as discussed in the main manuscript). There was no significant difference between the no interdependence condition and the interdependent with the target condition (model 1 *OR* = 1.11, *SE =* 0.11, *z* = 1.00, *p* = .948; model 2 *OR* = 1.09, *SE* = 0.11, *z* = 0.86, *p* > .999; model 3 *OR* = 1.09, *SE* = 0.11, *z* = -0.87, *p* > .999).

All three models showed a significant main effect of the target decision, post-hoc comparisons with a Bonferroni correction showed that gossip was more likely in the when the target cooperated than when the target defected (model 1 *OR* = 1.35, *SE* = 0.13, *z* = 3.28, *p* < .001; model 2 *OR* = 1.33, *SE* = 0.12, *z* = 3.08, *p* = .002; model 3 *OR* = 1.34, *SE* = 0.13, *z* = 3.12, *p=* .002).

Models 2 and 3 showed a significant effect of the round number, gossip was less likely in later rounds (model 2 *Est.* = -0.03, *SE* = 0.01, *z* = -5.09, *p* < .001; model 3 *Est.* = -0.03, *SE* = 0.01, *z* = -5.08, *p* < .001).

Model 3 showed a significant effect of gender, men were more likely to share gossip than women (*OR* = 1.79, *SE =* 0.41, *z* = -2.57, *p* = .010).

As in Study 1, we also compared within conditions whether engaging in gossip was more likely when the target defected or cooperated. In the interdependent with the target condition, gossip was not more or less likely when the target defected compared to when the target cooperated (Model 1 *OR* = 0.99, *SE* = 0.15, *z* = 0.07, *p* = .948; Model 2 *OR* = 1.01, *SE* = 0.15, *z* = 0.07, *p* = .943; Model 3 *OR* = 1.00, *SE* = 0.15, *z* = 0.02, *p* = .982). In the interdependent with the receiver condition, gossip was significantly more likely when the target defected compared to when the target cooperated (Model 1 *OR* = 2.19, *SE* = 0.38, *z* = 4.47, *p* < .001; Model 2 *OR* = 2.14, *SE* = 0.38, *z* = 4.30, *p* < .001; Model 3 *OR* = 2.14, *SE* = 0.38, *z* = 4.31, *p* < .001). In the no interdependence condition, gossip was not more or less likely when the target defected compared to when the target cooperated (Model 1 *OR* = 0.89, *SE* = 0.13, *z* = -0.80, *p* = .421; Model 2 *OR* = 0.89, *SE* = 0.13, *z* = -0.76, *p* = .450; Model 3 *OR* = 0.89, *SE* = 0.13, *z* = -0.78, *p* - .435).

**Table S10.**

*Models predicting the decision to gossip using interdependence structure conditions, the target decision of the target, and their interaction. Further models controlled for the round number, the data collection, age, and gender as independent variables (Study 2).*

|  | Model 1 | | Model 2 | | Model 3 | |
| --- | --- | --- | --- | --- | --- | --- |
| Predictor of decision to gossip | Wald χ^2^ | *p* | Wald χ^2^ | *p* | Wald χ^2^ | *p* |
| Condition | 70.16 | < .001 | 72.46 | < .001 | 72.19 | < .001 |
| Target decision | 19.98 | < .001 | 18.51 | < .001 | 18.57 | < .001 |
| Condition X Target decision | 12.89 | .002 | 12.45 | .002 | 12.28 | .002 |
| Round number |  |  | 25.92 | < .001 | 25.80 | < .001 |
| Data collection wave |  |  | 1.96 | .162 | 1.00 | .318 |
| Age |  |  |  |  | 0.09 | .763 |
| Gender |  |  |  |  | 6.59 | .010 |

*Note*. *df* = 2 for condition and condition X target decision, all other variables *df* = 1. Decision to gossip coded as 0 = refrain from gossip, 1 = gossip.

**Table S11.**

*All post-hoc comparisons for gossip decisions with a Bonferroni correction. Significant differences are in bold (Study 2).*

|  |  |  | Model 1 | | | | Model 2 | | | Model 3 | | |
| --- | --- | --- | --- | --- | --- | --- | --- | --- | --- | --- | --- | --- |
| Contrast decision to gossip |  |  | *OR* (*SE*) | 95% CI | *Z* | *p* | *OR* (*SE*) | *Z* | *p* | *OR* (*SE*) | *Z* | *p* |
| No interdependence & target cooperated | - | Interdependent w/ target & target cooperated | 0.95 (0.15) | [0.61; 1.49] | -0.31 | > .999 | 0.97 (0.15) | -0.17 | > .999 | 0.97 (0.15) | -0.19 | > .999 |
| **No interdependence & target cooperated** | **-** | **Interdependent w/ receiver & target cooperated** | **0.26 (0.05)** | **[0.15; 0.43]** | **-7.68** | **< .001** | **0.25 (0.04)** | **-7.73** | **< .001** | **0.25 (0.04)** | **-7.72** | **< .001** |
| No interdependence & target cooperated | - | No interdependence & target defected | 1.13 (0.17) | [0.73; 1.73] | 0.80 | > .999 | 1.12 (0.17) | 0.76 | > .999 | 1.12 (0.17) | 0.78 | > .999 |
| No interdependence & target cooperated | - | Interdependent w/ target & target defected | 0.96 (0.14) | [0.63; 1.48] | -0.26 | > .999 | 0.96 (0.14) | -0.25 | > .999 | 0.97 (0.14) | -0.22 | > .999 |
| **No interdependence & target cooperated** | **-** | **Interdependent w/ receiver & target defected** | **0.56 (0.08)** | **[0.36; 0.87]** | **-3.88** | **.002** | **0.53 (0.08)** | **-4.13** | **< .001** | **0.53 (0.08)** | **-4.12** | **< .001** |
| ***Interdependent w/ target & target cooperated** | **-** | **Interdependent w/ receiver & target cooperated** | **0.27 (0.05)** | **[0.16; 0.45]** | **-7.43** | **< .001** | **0.26 (0.05)** | **-7.64** | **< .001** | **0.26 (0.05)** | **-7.62** | **< .001** |
| Interdependent w/ target & target cooperated | - | No interdependence & target defected | 1.18 (0.17) | [0.77; 1.18] | 1.13 | > .999 | 1.15 (0.17) | 0.95 | > .999 | 1.16 (0.17) | 0.99 | > .999 |
| Interdependent w/ target & target cooperated | - | Interdependent w/ target & target defected | 1.01 (0.15) | [0.65; 1.56] | 0.07 | > .999 | 0.99 (0.15) | -0.07 | > .999 | 1 (0.15) | -0.02 | > .999 |
| ***Interdependent w/ target & target cooperated** | **-** | **Interdependent w/ receiver & target defected** | **0.59 (0.09)** | **[0.38; 0.91]** | **-3.56** | **.006** | **0.55 (0.08)** | **-3.98** | **.001** | **0.55 (0.08)** | **-3.95** | **.001** |
| **Interdependent w/ receiver & target cooperated** | **-** | **No interdependence & target defected** | **4.41 (0.76)** | **[2.67; 7.29]** | **8.66** | **< .001** | **4.49 (0.78)** | **8.68** | **< .001** | **4.50 (0.78)** | **8.70** | **< .001** |
| ***Interdependent w/ receiver & target cooperated** | **-** | **Interdependent w/ target & target defected** | **3.77 (0.65)** | **[2.27; 6.26]** | **7.70** | **< .001** | **3.86 (0.67)** | **7.81** | **< .001** | **3.88 (0.67)** | **7.83** | **< .001** |
| **Interdependent w/ receiver & target cooperated** | **-** | **Interdependent w/ receiver & target defected** | **2.19 (0.38)** | **[1.31; 3.66]** | **4.47** | **< .001** | **2.14 (0.38)** | **4.30** | **< .001** | **2.14 (0.38)** | **4.31** | **< .001** |
| No interdependence & target defected | - | Interdependent w/ target & target defected | 0.86 (0.12) | [0.57; 1.28] | -1.15 | > .999 | 0.86 (0.12) | -1.09 | > .999 | 0.86 (0.12) | -1.08 | > .999 |
| **No interdependence & target defected** | **-** | **Interdependent w/ receiver & target defected** | **0.50 (0.07)** | [0.33; 0.75] | **5.01** | **< .001** | **0.48 (0.07)** | **-5.24** | **< .001** | **0.48 (0.07)** | **-5.25** | **< .001** |
| ***Interdependent w/ target & target defected** | **-** | **Interdependent w/ receiver & target defected** | **0.58 (0.08)** | **[0.38; 0.89]** | **-3.87** | **.002** | **0.55 (0.08)** | **-4.19** | **< .001** | **0.55 (0.08)** | **-4.20** | **< .001** |

*Note*. Decision to gossip coded as 0 = refrain from gossip, 1 = gossip. *Note*. Please interpret comparison marked * with great caution because these conditions have different incentives for false gossip that could make the conditions incomparable as discussed in the main manuscript.

### Complete Models with Controls for Receiver Cooperation Study 2

Below we present an overview of models predicting whether receivers decided to cooperate (receiver decision) using the gossip content. Further models controlled for the round number, the data collection, age, and gender. Interpretation of significant main effects are presented below, see Table S12.

All three models showed a significant main effect of the gossip content, post-hoc comparisons with a Bonferroni correction showed that receivers were more likely to cooperate after receiving gossip that described the target as having cooperated than when no gossip was shared (model 1 *OR* = 1.91, *SE* = 0.20, *z* = 6.06, *p* < .001; model 2 *OR* = 1.88, *SE* = 0.20, *z* = 5.88, *p* < .001; model 3 *OR* = 1.88, *SE* = 0.20, *z* = 5.86, *p* < .001) and when gossip described targets as having defected (model 1 *OR* = 4.12, *SE* = 0.49, *z* = 11.96, *p* < .001; model 2 *OR* = 4.08, *SE* = 0.48, *z* = 11.86, *p* < .001; model 3 *OR* = 4.09, *SE* = 0.49, *z* = 11.87, *p* < .001). Receivers were less likely to cooperate after receiving gossip that described the target as having defected compared to when no gossip was receiving gossip (model 1 *OR* = 0.46, *SE =* 0.06, *z* = -6.34, *p* < .001; model 2 *OR* = 0.46, *SE* = 0.06, *z* = -6.38, *p* <.001; model 3 *OR* = 0.46, *SE =* 0.06, *z* = -6.42, *p* < .001).

Models 2 and 3 showed a significant effect of the data collection wave, receivers were less likely to cooperate (i.e., more likely to defect) in the second data collection wave in 2020 compared to the first data collection wave in 2019 (model 2 *OR* = 0.52, *SE* = 0.15, *z* = -2.20, *p* = .028; model 3 *OR* = 0.55, *SE* = 0.16, *z* = -2.05, *p* = .041).

Model 3 showed a significant effect of age, older receivers cooperated more (*Est.* = 0.17, *SE* = 0.06, *z* = 2.77, *p* = .006).

Comparing these results to the results of Study 1 shows that both studies found that people are more likely to cooperate if gossip described a target as having cooperated compared to having defected and that receivers are less likely to cooperate if a target is described as having defected versus not receiving gossip. Yet, Study 1 showed no difference between no gossip and gossip about cooperation while Study 2 showed that receivers who did not receive gossip were less likely to cooperate than receivers who received gossip that the target had cooperated. This could indicate that participants in Study 2 were generally less cooperative or that the incentives and real interactive setting of Study 2 (e.g., payment and only a single out of multiple rounds being paid) elicited more defection as the default behavior if no gossip was received because this could increase the chances of receiving the maximum bonus.

**Table S12.**

*Models predicting receiver decision to cooperate or defect using interdependence structure condition, the target decision of the target, and their interaction. Further models controlled for the round number, the data collection, age, and gender as independent variables (Study 2).*

|  | Model 1 | | Model 2 | | Model 3 | |
| --- | --- | --- | --- | --- | --- | --- |
| Predictor of receiver decision | Wald χ^2^ | *p* | Wald χ^2^ | *p* | Wald χ^2^ | *p* |
| Gossip | 144.24 | < .001 | 141.42 | < .001 | 141.65 | < .001 |
| Round number |  |  | 3.52 | .060 | 3.57 | .059 |
| Data collection wave |  |  | 4.86 | .028 | 4.18 | .041 |
| Age |  |  |  |  | 7.68 | .006 |
| Gender |  |  |  |  | 0.01 | .917 |

*Note*. *df* = 2 for condition and condition X target decision, all other variables *df* = 1. Receiver decision coded as 0 = defect, 1 = cooperate.

### Robustness Check Using Generalized Estimating Equations for Study 2 Hypotheses

We additionally used Generalized Estimating Equations to test hypothesis 1 (when a target defects, false gossip is more likely in the interdependent with the target condition compared to the no interdependence condition), hypothesis 2 (when a target cooperates, false gossip is more likely in the interdependent with the receiver condition compared to the no interdependence condition), and the additional comparison of false gossip when the target defected in the interdependent with the target condition with false gossip when the target cooperated in the interdependent with the receiver condition. The results are depicted in Table S13 for the full models and Table S14 for the contrast analyses regarding false gossip. Additionally, Figure S5 depicts the three-way interaction from Model 1 to illustrate the results of gossip and refraining from gossip across all conditions. All results were comparable to the GLMM results (i.e., true gossip was more likely than false gossip, gossip was more likely in the interdependent with receiver condition compared to other conditions, gossip about defection was more likely than gossip about cooperation, and men were more likely to gossip than women).

**Table S13.**

*Models predicting gossip veracity with interdependence structure condition, target decision, and their interaction. Further models controlled for the round number, the data collection, age, and gender as independent variables (Study 2).*

|  | Model 1 | | Model 2 | | Model 3 | |
| --- | --- | --- | --- | --- | --- | --- |
| Predictor of gossip occurring | Wald χ^2^ | *p* | Wald χ^2^ | *p* | Wald χ^2^ | *p* |
| Gossip veracity | 192.91 | < .001 | 193.84 | < .001 | 193.48 | < .001 |
| Condition | 54.43 | < .001 | 54.93 | < .001 | 52.34 | < .001 |
| Target decision | 23.07 | < .001 | 23.52 | < .001 | 22.22 | < .001 |
| Gossip veracity X Condition | 14.25 | < .001 | 14.21 | < .001 | 14.20 | < .001 |
| Gossip veracity X Target decision | 9.10 | .003 | 9.10 | .003 | 8.67 | .003 |
| Condition X Target decision | 10.21 | .006 | 10.50 | .005 | 11.83 | .003 |
| Gossip Veracity X Condition X Target Decision | 21.97 | < .001 | 22.19 | < .001 | 22.51 | < .001 |
| Data collection wave |  |  | 4.14 | .042 | 1.01 | .316 |
| Age |  |  |  |  | 2.71 | .100 |
| Gender |  |  |  |  | 48.22 | < .001 |

Note. *df* = 2 interactions, all other variables *df* = 1 Gossip occurring coded as 0 = did not occur, 1 = did occur.

**Table S14.**

*Contrasts testing hypothesis 1 (when a target defects, false gossip is more likely in the interdependent with the target condition compared to the no interdependence condition), hypothesis 2 (when a target cooperates, false gossip is more likely in the interdependent with the receiver condition compared to the no interdependence condition; Study 2).*

|  |  |  | Model 1 | | | Model 2 | | | Model 3 | | |
| --- | --- | --- | --- | --- | --- | --- | --- | --- | --- | --- | --- |
| Contrast false gossip occurred |  |  | *OR* (*SE*) | *Z* | *p* | *OR* (*SE*) | *Z* | *p* | *OR* (*SE*) | *Z* | *p* |
| Interdependent w/ target & target defected | - | No interdependence & target defected | 3.07 (0.45) | 7.65 | < .001 | 3.07 (0.45) | 7.62 | < .001 | 3.05 (0.45) | 7.54 | < .001 |
| Interdependent w/ receiver & target cooperated | - | No interdependence & target cooperated | 1.34 (0.33) | 1.19 | .236 | 1.34 (0.33) | 1.18 | .236 | 1.34 (0.33) | 1.19 | .233 |

**Figure S5.**

*The predicted probability to engage in true and false gossip per interdependence structure condition and target decision (Study 2).*

**

### Pre-registered Test of Interaction With Social Value Orientation (SVO)

We additionally pre-registered that participants with a proself SVO would be more likely to display self-serving false gossip in both the interdependent with target condition and the interdependent with receiver condition (vs the no interdependence condition). To test this, we used a generalized (binomial) mixed model with the interdependence structure condition (0 = no interdependence, 1= interdependent with the target, 2 = interdependent with the receiver), target decision (0 = defect, 1 = cooperate), social value orientation (0 = proself, 1 = prosocial) and all possible interactions as independent variables predicting gossip veracity (0 = true, 1= false).

SVO was measured using the SVO Slider Measure (Murphy et al., 2011). Participants indicated their preference for the division of money between themselves and an anonymous other across six items by selecting one out of nine divisions between the self and the other. Scores are calculated as an angle that represents an individual difference score at the ratio level based on the mean value allocated to the self and the other, higher scores indicate more prosociality. Scores ranged from 0° to 61.39° (M = 35.60°, SD = 11.83°). Making a dichotomous distinction between proself and prosocial resulted in 19 proself and 107 prosocial senders

Results showed no three-way interaction between the decision of the target, the interdependence structure condition, and SVO (χ^2^ (2) = 0.67, *p* = .714). Counter to the hypothesis, this indicates that the false gossip behavior did not depend on social value orientation. We found a similar result when using the continuous SVO Angle (higher angle is more prosocial; χ^2^(2) = 1.72, *p* = .433). We found similar results for our main analysis when including SVO as a covariate. In those models, we did find that (more) proself individuals were more likely to gossip falsely (dichotomous: *OR* = 2.15, *SE* = 0.65, *z* = 254, *p* = .011; angle: *Est*. (*SE*) = -0.03 (0.01), *z* = -2.89, *p* = .004 , *OR* = 0.98).

# References

Bates, D., Maechler, M., Bolker, B., & Walker, S. (2015). Fitting Linear Mixed-Effects Models Using lme4. *Journal of Statistical Software*, *67*(1), 1–48. https://doi.org/10.18637/jss.v067.i01

Clark, K., & Sefton, M. (2001). The sequential prisoner’s dilemma: Evidence on reciprocation. *The Economic Journal*, *111*(468), 51–68.

Constantin, A.-E., & Patil, I. (2021). ggsignif: R Package for Displaying Significance Brackets for “ggplot2.” *PsyArxiv*. https://doi.org/10.31234/osf.io/7awm6

Fox, J., & Weisberg, S. (2019). *An R Companion to Applied Regression* (Third). Sage. https://socialsciences.mcmaster.ca/jfox/Books/Companion/

Halekoh, U., Højsgaard, S., & Yan, J. (2006). The R Package geepack for Generalized Estimating Equations. *Journal of Statistical Software*, *15/2*, 1–11.

Lenth, R. (2021). *emmeans: Estimated Marginal Means, aka Least-Squares Means* (R package version 1.6.2-1) [Computer software]. https://CRAN.R-project.org/package=emmeans

Murphy, R. O., Ackermann, K. A., & Handgraaf, M. (2011). Measuring social value orientation. *Judgment and Decision Making*, *6*(8), 771–781.

R Core Team. (2021). *R: A Language and Environment for Statistical Computing* (4.1.2) [Computer software]. R Foundation for Statistical Computing. https://www.R-project.org/

Wickham, H. (2016). *ggplot2: Elegant Graphics for Data Analysis*. Springer-Verlag New York. https://ggplot2.tidyverse.org

Wilke, C. O. (2020). *cowplot: Streamlined Plot Theme and Plot Annotations for “ggplot2.”* https://CRAN.R-project.org/package=cowplot

1. We also pre-registered hypotheses regarding social value orientation, which we do not report here for brevity. There was no interaction between social value orientation (SVO), the interdependence structure condition, and the target’s decision (dichotomous SVO: χ^2^(1) = 0.19, *p* = 0.664; SVO angle: χ^2^(1) = 0.34, *p* = 0.562). [↑](#footnote-ref-1)
